# Reading without the left ventral occipito-temporal cortex

**DOI:** 10.1016/j.neuropsychologia.2012.09.030

**Published:** 2012-12

**Authors:** Mohamed L. Seghier, Nicholas H. Neufeld, Peter Zeidman, Alex P. Leff, Andrea Mechelli, Arjuna Nagendran, Jane M. Riddoch, Glyn W. Humphreys, Cathy J. Price

**Affiliations:** a Wellcome Trust Centre for Neuroimaging, Institute of Neurology, UCL, London WC1N 3BG, UK; b University of Toronto, Toronto, ON, Canada M5S 1A8; c Institute of Psychiatry, King’s College London, London SE5 8AF, UK; d School of Psychology, University of Birmingham, Edgbaston, Birmingham B15 2TT, UK; e Department of Experimental Psychology, Oxford University, Oxford OX3 9DU, UK

**Keywords:** Effective connectivity, Alexia, Recovery, Degeneracy, Word reading pathways, Functional MRI

## Abstract

The left ventral occipito-temporal cortex (LvOT) is thought to be essential for the rapid parallel letter processing that is required for skilled reading. Here we investigate whether rapid written word identification in skilled readers can be supported by neural pathways that do not involve LvOT. Hypotheses were derived from a stroke patient who acquired dyslexia following extensive LvOT damage. The patient followed a reading trajectory typical of that associated with pure alexia, re-gaining the ability to read aloud many words with declining performance as the length of words increased. Using functional MRI and dynamic causal modelling (DCM), we found that, when short (three to five letter) familiar words were read successfully, visual inputs to the patient’s occipital cortex were connected to left motor and premotor regions via activity in a central part of the left superior temporal sulcus (STS). The patient analysis therefore implied a left hemisphere “reading-without-LvOT” pathway that involved STS. We then investigated whether the same reading-without-LvOT pathway could be identified in 29 skilled readers and whether there was inter-subject variability in the degree to which skilled reading engaged LvOT. We found that functional connectivity in the reading-without-LvOT pathway was strongest in individuals who had the weakest functional connectivity in the LvOT pathway. This observation validates the findings of our patient’s case study. Our findings highlight the contribution of a left hemisphere reading pathway that is activated during the rapid identification of short familiar written words, particularly when LvOT is not involved. Preservation and use of this pathway may explain how patients are still able to read short words accurately when LvOT has been damaged.

## Highlights

► Word reading can succeed after damage to the left occipito-temporal cortex. ► This is enabled by an alternative pathway via the left superior temporal sulcus. ► Connectivity analysis demonstrated the existence of this pathway in normal readers. ► We hypothesise that this pathway integrates semantics with phonology. ► Our work stresses the importance of mapping alternative degenerate reading pathways.

## 1 Introduction

The importance of the left ventral occipito-temporal (LvOT) cortex for reading was first recognised on the basis of post mortem studies more than a century ago (Dejerine, 1891). Patients with damage to LvOT typically have impaired reading with relatively preserved writing (alexia without agraphia), speech and auditory language comprehension. This pattern of behaviour is referred to as “pure alexia” although, when tested extensively, difficulties with visual recognition of objects and colour naming are typically revealed (Starrfelt, Habekost, & Gerlach, 2010; Starrfelt, Habekost, & Leff, 2009). However, despite damage to LvOT, these patients can recover the ability to read short familiar words (e.g., Beeson, Magloire, & Robey, 2005; Henry et al., 2005; Ino et al., 2008; Tsapkini, Vindiola, & Rapp, 2011). This would suggest that there are neural pathways that can support rapid whole word identification without LvOT. The aim of the current study was to investigate *how* this is possible at the neural level. We refer to reading pathways that do and do not involve LvOT as the LvOT pathway and the reading-without-LvOT pathway.

We started our investigation with a brain imaging case study of a patient with extensive left occipito-temporal damage who was able to read short familiar words successfully under speeded conditions. When tested outside the scanner, the patient’s reading performance showed characteristics of pure alexia. She had profound reading difficulty which partially resolved and her reading performance was strongly influenced by word length and familiarity. Importantly, our functional imaging paradigm was specifically designed to focus on the neural pathways supporting accurate reading of rapidly presented short words, while minimizing the use of a serial letter processing strategy.

Areas that the patient activated when reading under speeded conditions were identified using functional magnetic resonance imaging (fMRI). Dynamic causal modelling (DCM) was then used to assess the strength and direction of functional connectivity between pairs of activated regions (Friston, Harrison, & Penny, 2003). Having identified the most likely reading-without-LvOT pathway in the patient, we tested whether the same reading-without-LvOT pathway was also activated by skilled readers performing exactly the same paradigm and, if so, how functional connectivity in the reading-without-LvOT-pathway was related to functional connectivity in the LvOT pathway. The analyses with skilled readers therefore allowed us to validate the findings from our patient’s case study.

Previous studies provide two contrasting predictions for possible reading-without-LvOT pathways. Functional imaging studies of patients with LvOT damage have suggested that the right vOT (RvOT) can support reading based on serial assimilation of letters (Cohen et al., 2004; Cohen et al., 2004; Gaillard et al., 2006; Henry et al., 2005; Ino et al., 2008; Leff et al., 2001; Pyun, Sohn, Jung, & Nam, 2007) but have not demonstrated how RvOT interacts with the language system or whether it can support rapid identification of short familiar words. In contrast, other studies have suggested that reading without LvOT depends on the strategy used (Coslett, 1996; Coslett, Saffran, Greenbaum, & Schwartz, 1993) and can be sustained by compensatory activations in the superior parietal lobule that might be involved in phonological working memory (Henry et al., 2005; Ino et al., 2008) or the anterior middle temporal gyrus that might be involved in accessing the semantic system (Tsapkini et al., 2011). Although a few studies have suggested that other parallel reading pathways may connect visual occipital areas to temporal or parietal regions without necessarily involving LvOT (Henderson, 1986; Iwata, 1984; Levy et al., 2009; Richardson, Seghier, Leff, Thomas, & Price, 2011; Sakurai, 2004), the neural basis of this alternative pathway remains unknown. Here, we hypothesized that such a reading-without-LvOT pathway might involve functional connectivity between the left inferior occipital cortex and the left superior temporal sulcus. This hypothesis was based on a recent study of skilled readers that found evidence for two different but concurrently activated pathways linking the left inferior occipital cortex to the posterior superior temporal sulcus: one involving LvOT and one without LvOT (Richardson et al., 2011). The current study of a pure alexic and 29 healthy skilled readers investigated whether reading-without-LvOT pathway(s) can support reading in the absence of detectable activation in the LvOT pathway(s).

## 2 Materials and methods

This study was approved by the National Hospital for Neurology and Neurosurgery and Institute of Neurology Joint Ethics Committee. All subjects gave written informed consent.

### 2.1 Subjects

A number of 29 healthy right handed native English speakers (16 females, 13 males, aged 33±18 years) with no history of neurological or psychiatric disorders participated in our study. All had normal or corrected-to-normal vision, and activated all our regions of interest during the functional imaging paradigm (see below for details).

### 2.2 Patient

**Fig. 1:**
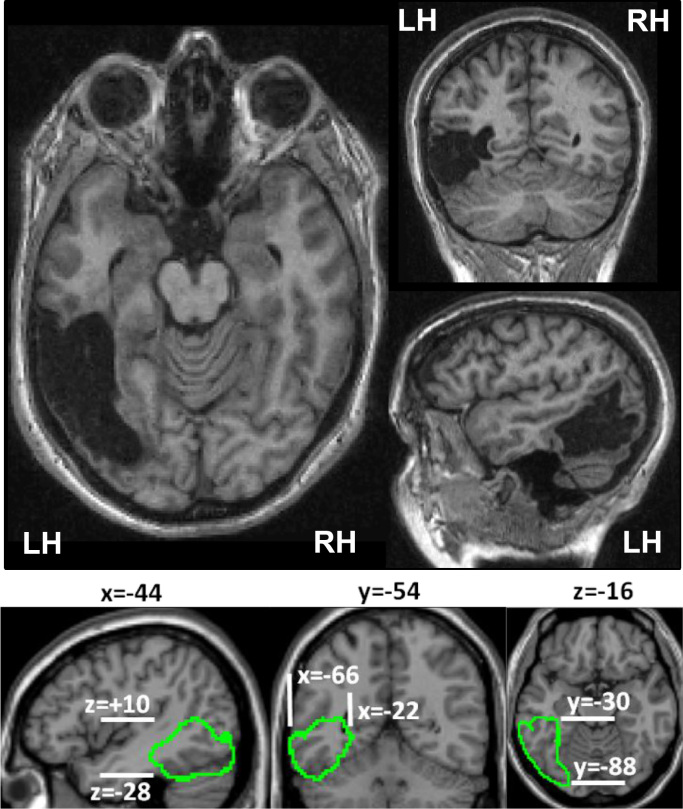
(Top) Illustrates the left occipito-temporal lesion in AH. The lesion is shown in axial, coronal and sagittal views of the raw high-resolution T1-weighted image. (Bottom) The extent of AH’s lesion in the MNI space is shown on three different views.

Our patient, AH, is a right-handed 49-year-old female who presented with a headache and visual disturbances following a venous thrombosis that led to secondary haemorrhage. Orthoptic investigation confirmed a small bilateral right superior visual field loss which mildly improved over the first three months after stroke (see Fig. S1 of the Supplementary material). Speech production, comprehension, and repetition were normal. No other deficits were apparent in the patient at 4 years after stroke. Structural MRI identified a large lesion in the left occipito-temporal region. Automated lesion identification (Seghier, Ramlackhansingh, Crinion, Leff, & Price, 2008) showed that AH’s lesion covered a cerebral volume of 41 cm^3^ (a volume roughly equivalent to that of a golf ball). This lesion extended in MNI space from −22 mm to −66 mm in the *x*-axis, from −88 mm to −30 mm in the y axis and from −28 mm to +10 mm in the *z* axis (Fig. 1).

#### 2.2.1 The patient’s reading and letter naming performance (out of scanner)

The left occipito-temporal damage severely impaired the patient’s ability to read. Her writing was relatively preserved as examined with a written picture description task at 5 months after stroke (see Fig. S2 of the Supplementary material). When making errors in the written picture description task, the patient subsequently made self-corrections consistent with a diagnosis of alexia without agraphia. Her comprehension of short familiar written words was preserved (see example of her performance during a semantic association task in Fig. S3 of the Supplementary material). She was better able to read short rather than long words (see below for details) and often named individual letters prior to producing the whole word (consistent with a “letter-by-letter” reading strategy). Below we report the assessment of her reading that was conducted nearly 4 years after her stroke (46 months) at the time of the fMRI study. In each task, stimuli were presented in point 18 Times New Roman font, with stimulus presentation managed by E-Prime software (http://www.pstnet.com; Psychology Software Tools, Inc., PA-Sharpsburg). Experiments were conducted on a Toshiba laptop computer and vocal reaction times were recorded using an attached voice-key.

#### 2.2.2 Letters and numbers

**Fig. 2:**
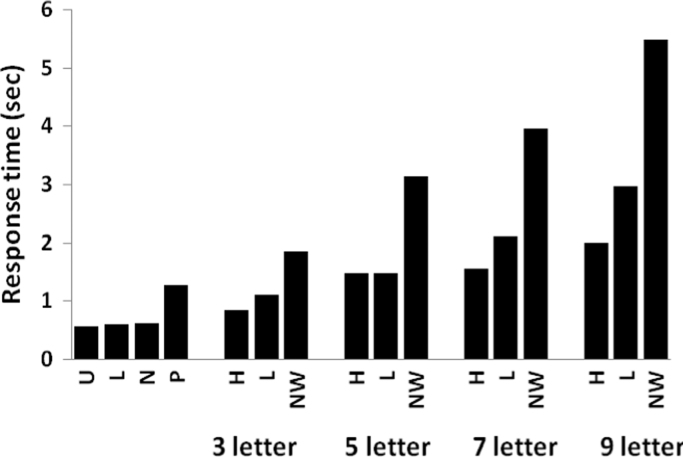
Out-scanner responses times [in seconds] in the patient for lower-case (L) and upper-case (U) letters, numbers (*N*), pictures (P), high frequency (*H*) and low frequency (*L*) words, and nonwords (NW). Word and nonword conditions were tested at different word lengths (3 to 9 letters).

Each letter of the alphabet (26 lower-case and 26 upper-case) and the numbers 2 through 9 were presented three times each in a pseudorandom order. The patient correctly named 76/78 lower-case letters, 76/78 upper-case letters and 24/24 numbers. Her errors (e.g., /b/ identified as D on one trial and /d/ on another; and E identified as F) were typical of those made by pure alexic patients (Fiset, Arguin, Bub, Humphreys, & Riddoch, 2005). The mean response times for naming individual letters was 570 ms after removing trials in which the voice-key failed to trigger and outlier values that exceeded two standard deviations from their condition mean (Fig. 2).

#### 2.2.3 Word reading

AH was presented with a total of 160 words that manipulated the factors of length (three, five, seven, nine letters) and frequency (high, low). Words were presented in pseudorandom order. The patient made a total of six errors, which were distributed across the various experimental conditions. Correct RTs were analysed by means of a two-way ANOVA treating each RT as an independent subject with the factors being length (three, five, seven, nine) and frequency (high, low). The results demonstrated main effects of length (*F*(3, 139)=24.129, *p*<0.001) and frequency (*F*(1, 139)=11.692, *p*=0.001) and a significant length×frequency interaction (*F*(3, 139)=2.66, *p*=0.05) (see Fig. 2 for details). The effect of frequency (low>high) was only significant for nine-letter words (*t*(36)=2.635, *p*=0.012). In summary, the patient showed a word length effect requiring approximately 280 ms per letter, consistent with the letter-by-letter reading strategy that typically follows LvOT damage (Cohen et al., 2004; Henry et al., 2005; Leff, Spitsyna, Plant, & Wise, 2006; Upton, Hodgson, Plant, Wise, & Leff, 2003). However, a word length effect of 280 ms per letter is moderate (e.g., Behrmann, Plaut, & Nelson, 1998; Hanley & Kay, 1996; Patterson & Kay, 1982) and AH was still able to read three letter words in approximately 1 s. This suggests that AH had some preserved parallel letter processing abilities despite the loss of her LvOT (Tsapkini & Rapp, 2010). During the scanning sessions (see below) she was given an average of 1.5 s to read each 3–6 letter word; this relatively fast presentation rate encouraged the patient and controls to read as rapidly as possible while minimizing the likelihood that the patient would adopt an overt letter-by-letter reading strategy.

#### 2.2.4 Nonword reading

The patient was presented 160 nonwords that varied in length (three, five, seven, nine) and nonword ‘familiarity’ (familiar, unfamiliar). ’Familiar’ nonwords included items such as *toredom, meeking* and *hospile* that are only one letter away from a real word, whilst *smazung*, *twavcla* and *clajelb* were amongst the ‘unfamiliar’ nonwords because they were not orthographically close to real words. Items were presented in pseudorandom order. Reading accuracy was analysed by means of a log-linear analysis, with factors of length (three, five, seven, nine), nonword familiarity (familiar, unfamiliar) and accuracy (correct, incorrect). Results demonstrated significant interactions between length and accuracy (*χ*²(3)=13.206, *p*=0.0042), reflecting improved reading of shorter nonwords compared to longer nonwords (three-letters=33/40, five-letters=28/37, seven-letters=30/39, nine-letters=18/38), and between familiarity and accuracy (*χ*²(1)=7.733, *p*=0.0054), demonstrating significantly better reading of ‘familiar’ nonwords (63/78) than ‘unfamiliar’ items (46/75). Correct RTs were analysed by means of a two-way ANOVA, with factors of length (three, five, seven, nine) and nonword familiarity (familiar, unfamiliar). There were reliable main effects of length (*F*(3, 96)=47.304, *p*<0.001) and familiarity (*F*(1, 96)=24.019, *p*<0.001) but no significant length×familiarity interaction (*F*(3, 96)=1.669, n.s.), see Fig. 2 for details. The main effect of familiarity was a consequence of significantly faster naming for familiar (3094.7 ms) compared to unfamiliar (4131.7 ms) nonwords. This effect of familiarity indicates the influence of lexical and/or sublexical knowledge on nonword decoding.

#### 2.2.5 Exception word reading

The patient was tested on a set of 150 words used by Mechelli and colleagues to explore how neuronal interactions within the reading system are influenced by word type (Mechelli et al., 2005). Words varied in length (between 4 to 9 letters in length) and they included (i) 75 regular words with typical spelling-sound correspondences (e.g., cult, shock, fact, chill), and (ii) 75 exception words with atypical/inconsistent spelling-sound correspondences (e.g., folk, debt, doubt, cough). Words were visually presented for 3 s with a stimulus onset asynchrony of 4 s. All words were matched for number of letters, number of syllables, bigram frequency, familiarity, imageability, and written frequency that ranged from 1 to 447 (per million) with a mean of 40.8 and a standard deviation of 66.48; for more details see (Mechelli et al., 2005). For regular words, the patient was able to read 48 out of 50 (96%) of words with 4–6 letters in length and 18 out of 25 (72%) of words with 6–9 letters in length. All errors were null responses except for “enigma” which she said “elephant”. For exception words, the patient was able to read 41 out of 50 (82%) of words with 4–6 letters in length and 15 out of 25 (60%) of words with 6–9 letters in length. All errors were null responses except for “myth” which she said “my” followed by “th”and for “parachute” which she said “para” followed by “sh” (i.e., regularization errors). In sum, irrespective of word length, the patient successfully read 75% of exception words that were visually presented for 3 s.

#### 2.2.6 Picture naming abilities

Given unlimited presentation time, the patient was able to name 70/76 pictures from the Birmingham Object Recognition Battery (BORB) (Riddoch & Humphreys, 1993). Under speeded conditions (a triad of three pictures per 4.5 s) the patient’s performance was only 48% over a total set of 32 triads of pictures of familiar objects. This is consistent with previous studies of patients with LvOT damage, although picture naming can appear to be normal at unlimited exposure durations, errors occur when exposure duration is reduced (Friedman & Alexander, 1984; Starrfelt et al., 2009).

#### 2.2.7 Summary of the patient’s reading ability

As detailed above, the patient’s reading was not consistent with either phonological or surface dyslexia. Rather, the patient’s reading followed, in an exaggerated way, the same pattern that would be expected from normal subjects. Reading difficulty was more apparent for unfamiliar nonwords and words with atypical spellings when compared with words with typical spelling (e.g., Binder, Medler, Desai, Conant, & Liebenthal, 2005; Fiez, Balota, Raichle, & Petersen, 1999).

### 2.3 Functional imaging paradigm

The aim of the out-of-scanner testing reported above was to establish the limits of the patient’s reading ability and to demonstrate the word length effect, on errors and response times, as typically observed following LvOT damage. The aim of the in-scanner testing was to compare the neural pathways that could be used to support accurate and relatively fast reading of short familiar words, when the stimulus duration and stimulus onset asynchrony were held constant in the patient and controls. The stimuli for the fMRI experiment were therefore short familiar object names with high word frequency. Most of the object names had three, four or five letters. Longer object names (e.g., elephant, kangaroo, saucepan, wardrobe) were deliberately removed from the stimulus lists. The patient also participated in an fMRI study of reading that manipulated the length of words and pseudowords using the same experimental designs that Mechelli et al. (2005) reported with data from healthy controls. However, the patient was unable to keep up with the demands of this paradigm and made so many mistakes that her activation pattern was rendered meaningless (Price & Friston, 1999). We therefore only report the patient’s neuronal activation from the current paradigm with short high frequency words. Moreover, by focusing on activation for correct trials only, we compare the patient to controls who are producing exactly the same response. Even here, it is likely that the reading strategy used by the patients differed from some or all of the controls. We used our standard language activation paradigm that has been reported several times before (Josse, Seghier, Kherif, & Price, 2008; Seghier, Fagan, & Price, 2010). It involves four conditions with written words or pictures of objects (reading aloud; naming pictures aloud; semantic decisions on written words; and semantic decisions on pictures of objects) and four sensori-motor baseline conditions (saying 1, 2, 3 to meaningless Greek letter strings; saying 1, 2, 3 to pictures of meaningless nonobjects; perceptual decisions on Greek letters; and perceptual decisions on nonobjects). This experimental paradigm allowed us to identify the network of regions that were activated by reading aloud relative to blocks of fixation (see below) and then identify their function according to their activation profile across the eight conditions as detailed below.

### 2.4 Stimuli and presentation parameters

All stimuli were derived from a set of 192 objects with three to six letter names and regular spelling to sound relationships: 33 objects had three letter names (cat, bus, hat), 65 had four letter names (ship, bell, frog, hand), 58 had five letter names (teeth, camel, snake) and 36 had six letter names (spider, dagger, button). In all trials for all conditions, three stimuli were simultaneously presented as a “triad”, with one stimulus above and two stimuli below. The interval between the onset of each triad was always 4.5 s. Only trials where all three stimuli in a triad were read correctly were considered as correct trials. Stimuli were presented via a video projector, a front-projection screen and a system of mirrors fastened to a head coil. The four conditions requiring an articulation response were presented in scanning sessions separate from the semantic and perceptual conditions requiring a finger press response. There were four blocks of written words, four blocks of pictures, two blocks of Greek letters and two blocks of nonobjects in each session. Each block lasted 18 s with 12 stimuli per block that were presented in four triads with a duration of 4.32 s per triad and an inter-stimulus interval of 4.5 s. Each block was preceded by 3.6 s of instructions (e.g., READ) and the patient was able to read and understand the written instructions. Fixation blocks (14.4 s) were then interleaved and presented after every two condition blocks (total six blocks of fixation).

For the reading and object naming conditions, triads of stimuli were constructed such that there was no obvious semantic relationship between the three different items in the triad (e.g., slide, axe, cup). Accuracy of vocal responses was recorded with a MRI-compatible microphone. Subjects were instructed to whisper their responses with minimal mouth movement to minimize artefact from head motion and airflow. A sound cancellation system allowed for identification of accurate vocal responses however the quality of the speech recordings was not sufficient to extract response times.

In the semantic conditions, subjects were required to indicate (with a left or right finger press) which one of the two choices on the bottom of the triad was most semantically related to the target on the top of the triad. In the perceptual conditions, one of the items on the bottom of the triad was perceptually identical to the target item above and subjects were instructed to indicate which of the two choices looked identical to the target. Responses for the semantic and perceptual tasks were made with the index and middle fingers on a key pad to measure accuracy and response times.

### 2.5 The patient’s in-scanner responses during reading aloud

The triads in our paradigm were presented every 4.5 s with a duration of 4.32 s per triad (=1.44 s per word). This relatively fast stimulus presentation rate encouraged the patient and healthy subjects to respond as rapidly as possible and ensured that the words in a triad could not be read using an overt letter by letter reading strategy. Our rationale here was to discourage the patient from relying too heavily on serial letter identification thereby minimising the differences in how she and the controls were reading. Put another way, the speeded conditions of the same stimuli ensured relatively comparable reading between the patient and controls, albeit not necessarily with the same ability. We could then focus on differences in the neural activation in (a) the patients and controls; and (b) within the controls.

Behavioural responses for our patient were recorded under speeded conditions and short high frequency stimuli were read aloud with good accuracy (71.9%). The majority of incorrect items were attributable to missed responses to one word in the triad. Although it was not possible to measure AH’s reading speed in the scanner, we can infer her speed was relatively good as she was able to correctly read all three words in nearly half of all (15/32) triads. Although she sometimes resorted to an overt letter-by-letter reading strategy for unfamiliar words read outside the scanner, our in-scanner recordings of her speech confirmed she did not rely on an overt letter-by-letter reading strategy whilst being scanned. However, the word length effect that was apparent in the out of scanner tasks (approximately 280 ms per letter) indicates that the patient may have been more reliant on a serial reading strategy than the controls. Fast implicit reading and access to word meaning was evidenced by her ability to make semantic decisions on words with high accuracy (80%) (e.g., Coslett & Saffran, 1989).

### 2.6 MRI acquisition

Experiments were performed on a 1.5T Siemens system (Siemens Medical Systems, Erlangen, Germany). Functional imaging consisted of an EPI GRE sequence (TR/TE/Flip=3600 ms/50 ms/90°, FOV=192 mm, matrix=64×64, 40 axial slices, 2 mm thick with 1 mm gap). Functional scanning was always preceded by 14.4 s of dummy scans to ensure tissue steady-state magnetization. Anatomical T1-weighted images were acquired using a three-dimensional modified driven equilibrium Fourier transform sequence (TR/TE/TI=12.24 ms/3.56 ms/530 ms, matrix=256×224, 176 sagittal slices with a final resolution of 1 mm^3^).

### 2.7 fMRI data analysis

Data processing and statistical analyses were performed with the Statistical Parametric Mapping SPM5 software package (Wellcome Trust Centre for Neuroimaging, London UK, http://www.fil.ion.ucl.ac.uk/spm). All functional volumes were spatially realigned, un-warped, normalized to the MNI space using the unified normalisation–segmentation procedure of SPM5, and smoothed with an isotropic 6 mm FWHM Gaussian kernel, with a resulting voxel size of 2×2×2 mm^3^. Time-series from each voxel were high-pass filtered (1/128 Hz cut-off) to remove low-frequency noise and signal drift. The pre-processed functional volumes of each subject were then submitted to a fixed-effects analysis, using the general linear model at each voxel. Each stimulus onset for correct trials was modelled as an event in condition-specific ‘stick-functions’ with a duration of 4.32 s per trial and a stimulus onset interval of 4.5 s. Correct responses for each condition, instructions, and errors were modelled separately in the design matrix. The resulting stimulus functions were convolved with a canonical hemodynamic response function which provided regressors for the linear model. Eight contrast images, one for each of the eight conditions relative to fixation, were generated for each subject. These contrast images were subsequently included in second-level analyses to compare correct responses to the different conditions as detailed below. Errors were excluded from second level analyses.

### 2.8 Region selection

**Fig. 3:**
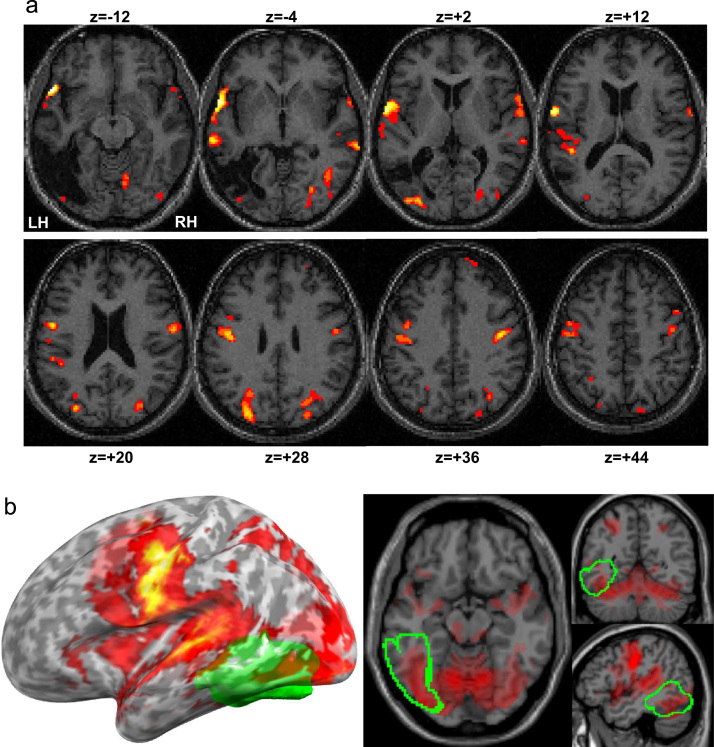
(A) Illustrates the activations in our patient for reading aloud relative to fixation, at *p*<0.05 corrected for height or extent. All activated clusters are shown in red-to-yellow colour scale and projected on axial slices of the anatomical scan of the patient, varying from *z*=−12 mm to *z*=+44 mm. (B) Illustrates the overlap between the activated pattern (*p*<0.05 corrected) during reading aloud of the healthy controls (red-to-yellow colour scale) with the damaged region in the patient (green). A large part of the ventral visual activations in the healthy controls overlaps with the patient’s lesion (outlined in green).

Our connectivity analyses included regions that were more activated during reading aloud in the patient compared with our 29 normal readers (two sample *t*-test, reported at *p*<0.05 following family wise error (FWE) correction for multiple comparisons in height) to identify reading pathways that might compensate for loss of LvOT. In total there were 15 regions of interest (see Table 1 for a full list of coordinates), including ventral and dorsal occipital regions, ventral occipito-temporal cortex, motor and ventral premotor cortex, globus pallidus, planum temporale and superior temporal sulcus (Table 1 and Fig. 3). To identify the functional responses associated with the regions activated during reading, a second-level analysis with all eight conditions was carried out in the normal readers. From this analysis, (i) visual areas were activated in the main effect of all eight conditions (*p*<0.05 FWE-correction for multiple comparisons in height), inclusively masked with eight individual contrasts (thresholded at *p*<0.001 uncorrected) that pertained to each condition relative to fixation, and (ii) articulation areas were those that were activated by reading, naming and saying 1, 2, 3 relative to the semantic and perceptual conditions that involved a finger press response (*p*<0.05 FWE-corrected for multiple comparisons in height). See Fig. S4 of the Supplementary material for more details.

#### 2.8.1 Occipital regions

Visual processing regions were identified in the left and right ventral and dorsal quadrants of the occipital lobe. We abbreviate the names of these areas to: left vOCC, right vOCC, left dOCC and right dOCC. The increase in activation in posterior visual areas is in line with a previous fMRI study of developmental dyslexia that showed abnormally strong involvement of visual areas in the context of abnormally weak activation in LvOT (Wimmer et al., 2010).

#### 2.8.2 Frontal regions

Within the large sensori-motor and auditory pattern seen in Fig. 3 and Fig. S4 of the Supplementary material, articulation areas in the frontal lobe with higher reading activation in the patient than normal readers were identified in the left ventral premotor cortex (left vPM) and the left central sulcus (including the motor cortex (left M)).

#### 2.8.3 Temporal regions

Two left temporal regions showed significantly greater reading activation in the patient than the normal readers, including a left planum temporale (left PT) region associated with auditory feedback during articulation (Guenther, Ghosh, & Tourville, 2006) and a central part of the left superior temporal sulcus (left STS) that has recently been associated with the integration of semantics and phonology (Binder & Desai, 2011; Richardson, Thomas, & Price, 2010); see Fig. S5 of the Supplementary material for an illustration of the activation profile of these temporal regions.

#### 2.8.4 Left ventral occipito-temporal cortex

As the patient’s lesion meant that she lacked activity in LvOT, we searched within the F-map for LvOT voxels where the effect of reading was greatest. Signal from these voxels was well below the threshold for significance and was expected to be noise used here as a realistic approximation of a dysfunctional LvOT in our connectivity models. By including LvOT in our DCM analysis we were able to compare the contribution of this region in the patient and controls.

#### 2.8.5 Other left hemisphere regions

The patient activated other reading areas within a normal range (i.e., patient>controls not significant), including the anterior cingulate, left superior parietal lobule and bilateral cerebellum. These regions were not included in our DCM analyses.

#### 2.8.6 Right hemisphere regions

The globus pallidus external (GPe) was the only other area where the patient showed greater reading activation than controls in the right hemisphere. This was categorised as an articulation area in the patient (similarly activated by reading, naming and saying “1,2,3” relative to semantic and perceptual tasks), consistent with the corresponding left hemisphere homologue of this area being significantly activated by articulation in the control subjects (see Table 1). Finally, we also considered the contribution of right vOT, right STS, right PT, right vPM and right M in our DCM analyses in the patient. These areas were activated in the patient but not more strongly than controls (at *p*<0.001 uncorrected).

### 2.9 Data extraction from each of the selected regions

Our DCM analyses only included data from the “articulation” sessions that involved reading aloud, naming and saying 1, 2, 3 since our aim was to identify pathways supporting reading aloud in the absence of LvOT. In each subject, eigenvectors (i.e., time series) were separately extracted from our 15 regions of interest for the 2 articulation sessions and adjusted to the *F*-contrast of each subject. Data from the two sessions were then concatenated prior to being incorporated in the DCM model (Seghier & Price, 2010). In the patient, the eigenvectors were extracted from a 4 mm-radius sphere centred at the co-ordinates showing the most significant increase in activation relative to the controls (see Table 1). For each control, eigenvectors were extracted from a 4 mm-radius sphere centred at the co-ordinates where activation was highest within a 6 mm-distance of that extracted from the patient.

### 2.10 Modelling effective connectivity with DCM

Briefly, DCM is a hypothesis-driven neurodynamics model that uses a bilinear state equation to characterise an experimentally perturbed cognitive system (Friston et al., 2003). After defining a model with a set of regions and connections, DCM estimates the different parameters of this model at the neuronal level using a hemodynamic forward model. DCM then compares the generated/modelled functional responses to the measured ones (i.e., the extracted time series). Thanks to its biophysical forward model of hemodynamic responses, the main advantage of DCM is the opportunity to infer mechanisms at the neuronal level which provides a more precise estimation of how the rate of change of activity in one region influences the rate of change in other regions. This in turn leads to information about the direction of the influence one brain region may have on another rather than implying a non-directional correlation. More details about DCM can be found elsewhere (e.g., Seghier, Zeidman, Neufeld, Leff, & Price, 2010; Stephan et al., 2010).

### 2.11 Connectivity parameters in DCM

For a given model, DCM estimates three different sets of effective connectivity parameters: (i) input parameters that quantify how brain regions respond to external stimuli, (ii) endogenous parameters reflecting the average or baseline connectivity that characterises the coupling between regions in the absence of external inputs, and (iii) modulatory parameters that measure changes in effective connectivity induced by experimental conditions. These different parameters are expressed in Hz within the DCM framework. We defined the following inputs in our DCM models: (i) all visual triads with correct responses were grouped as a single driving input that entered the system at specific driving region(s), (ii) all correct trials with meaningful stimuli “naming+reading” were defined as a first modulatory input/context; and (iii) their difference “naming–reading” was defined as a second modulatory input.

As detailed below, our DCM analyses were carried out separately on patient and control data. DCM of the patient data involved the following analyses: (i) we first identified the best driving occipital region after comparing the model evidence for eight different models that included four occipital regions, (ii) within a DCM model space of 124 plausible models, we identified the best model that explained the patient’s significant activation in the five left hemisphere regions reported in Table 1, (iii) the patient’s dysfunctional LvOT was connected to the winning model in step (ii) to test how the model evidence changed as the number of connections to and from a dysfunctional LvOT increased. This resulted in the comparison of 31 models with 6 regions. Finally, (iv) we investigated the contribution of the right hemisphere in a set of five different models that varied in the number of regions and connections. For the controls, DCM analyses involved the same 6-region model as in step (iii) with the patient. The aim was to test how the model evidence changed as the number of connections to and from an intact LvOT increased. Finally, for the 6-region model, we investigated how the connection strengths varied between the patient and controls and within the controls. The aim here was to dissociate alternative neuronal pathways for the same reading task.

### 2.12 DCM analyses of the patient data

It is currently not feasible to explore all possible combinations of inter-regional connections within a 15 region and 210 connection model since the possible combinatorics of inter-regional connections rises exponentially with the number of regions in a model. We therefore investigated the model space in a step by step fashion (Stephan et al., 2010).

#### 2.12.1 Step 1: Which visual processing area is the input to the rest of the system in the patient?

**Fig. 4:**
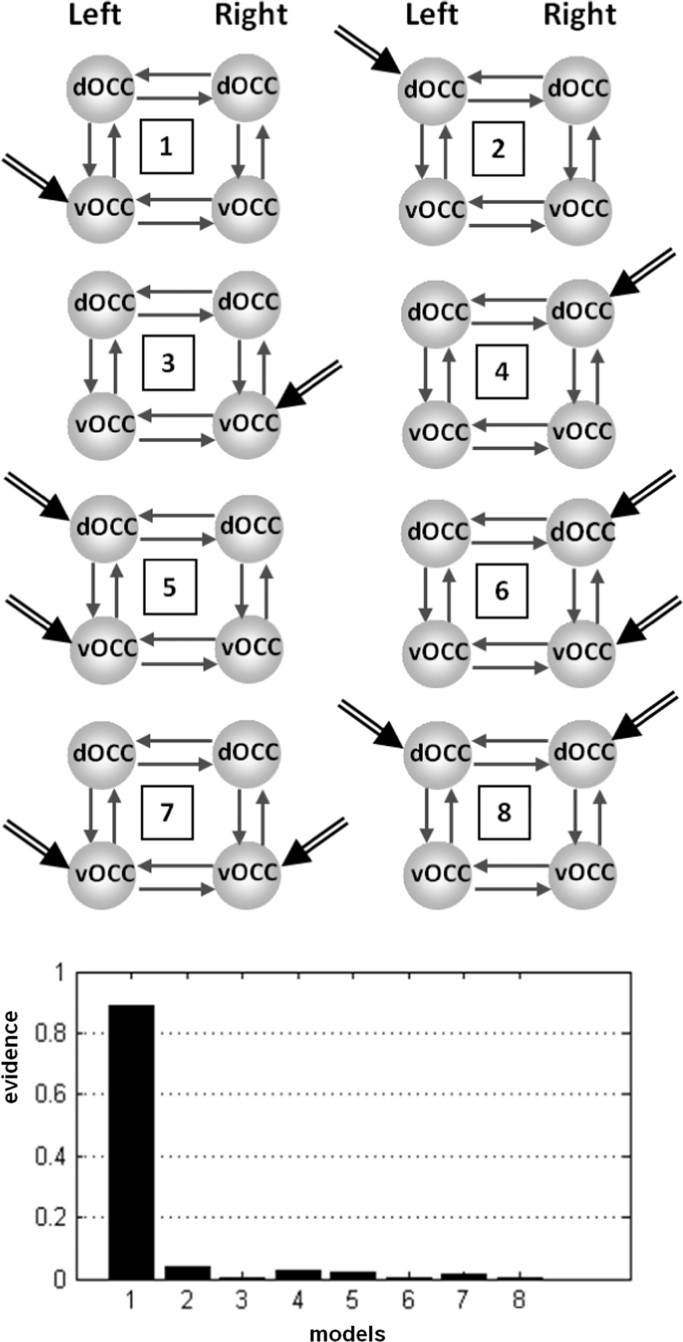
BMS analysis over eight models with four occipital regions in the patient that varied in where the driving inputs entered the DCM models. The best model is the one with the highest model evidence (see posterior model probabilities in the bar graph), showing strong evidence for a left vOCC driving region. vOCC=ventral occipital region; dOCC=dorsal occipital region.

Here we established which of the visual processing regions (left vOCC, left dOCC, right vOCC, right dOCC) should be used as the input area(s) to the rest of the reading system. This involved a comparison of the model evidence (see below for analysis details) for eight different models which each had four regions but differed in the location of the input region (Fig. 4). Having established that left vOCC propagated activation throughout the system, all further models included left vOCC as their input region (for a similar rationale see Penny et al. (2010), Seghier, Josse, Leff, & Price, 2011).

#### 2.12.2 Step 2: Which set of connections best explains the patient’s data in the left hemisphere 5-region model?

This step concerned the five left hemisphere regions that showed stronger activation in the patient than controls (*p*<0.05 FWE-corrected). Specifically, we investigated how visual information passed from the visual input region to the left hemisphere articulation areas by comparing the evidence of all possible models in the patient that included the five intact left hemisphere regions (i.e., vOCC, STS, PT, vPM and M), with vOCC as the input region, but differed in the presence or absence of connections between regions. We assumed that visual inputs were propagated from the vOCC to the articulation areas (vPM and M) via either STS or PT. Therefore we did not include models that were missing both the vOCC to STS and vOCC to PT connections; and none of our models included direct connections from vOCC to vPM/M. This yielded a total of 124 possible models.

#### 2.12.3 Step 3: How does evidence for the 5 region model in the patient change when it is connected to LvOT?

Here we investigated the contribution of LvOT by adding this region to the winning model from Step 2 to create a 6 region model. Evidence was compared for all possible combinations of functional connections to and from LvOT and the five other regions. This yielded a total of 31 models and allowed us to test how the model evidence changed as the number of connections with LvOT increased.

#### 2.12.4 Step 4: Does the right hemisphere contribute in the patient?

Here we investigated the contribution of the right hemisphere in the patient. Assuming homologue pathways to those in the left hemisphere, we tested the significance of the right hemisphere inter-regional connections in each of three different models. Model A included data from the six right hemisphere regions that were the homologues of the six left hemisphere regions included in Step 3 above, with right vOCC as a driving region; for a similar procedure see (Acs & Greenlee, 2008). Model B was the same as Model A with the inclusion of right GPe. Model C was the same as Model A but also included the six left hemisphere homologues. The latter resulted in a large 12 region model in which plausible inter-hemispheric interactions were limited solely to homotopic connections. There were three versions of Model C with inputs to left vOCC, right vOCC and both left/right vOCCs. The robustness of DCM for assessing inter-hemispheric connectivity has been demonstrated in many studies, for instance during visual integration (Stephan, Marshall, Penny, Friston, & Fink, 2007), audiovisual emotion processing (Müller, Cieslik, Turetsky, & Eickhoff, 2012), hand movement coordination (Grefkes, Eickhoff, Nowak, Dafotakis, & Fink, 2008), reading aloud (Carreiras et al., 2009), semantic decisions (Seghier et al., 2011), and phonological processing (Bitan, Lifshitz, Brenitz, & Booth, 2010).

### 2.13 DCM analyses of control data

The analyses in normal readers were based on the six left hemisphere region models investigated in Step 3. All six regions were significantly activated in our healthy controls and were extracted at the nearest local peak of each individual subject to the coordinates of the patient (see full list of all subject-specific coordinates in Table S1 of the Supplementary material). We generated 31 models for each control that varied in the number of connections with LvOT. First, as in Step 3 of the patient analyses above, we tested how the model evidence was impacted by increasing the number of connections with LvOT. Second, we compared the connection strength in controls and the patient for each connection in the 6 region model (a total of 26 interregional connections).

### 2.14 Inter-subject variability in the reading pathways used by controls

The aim of this analysis was to determine whether healthy skilled readers differed from one another in their relative reliance on the pathways connecting vOCC to the articulation areas (vPM and M). To find structure in the inter-subject variability of endogenous connectivity (i.e., how variance in one connection is related to variance in another), we used an agglomerative hierarchical clustering algorithm (Hastie, Tibshirani, & Friedman, 2009), the hyperbolic correlation as a dissimilarity metric (Golay et al., 1998; Seghier & Price, 2009), and complete linkage clustering as a linkage criteria (Hastie et al., 2009). The rationale for this unconstrained clustering approach was to group together the connections that increased or decreased similarly across subjects and examine whether there were dissociations between pathways across subjects. Correlations between connectivity parameters have previously been used, for instance to assess the relationship between input and output cerebellar connections during a rhyming judgement task (Booth, Wood, Lu, Houk, & Bitan, 2007).

### 2.15 Implementation of the DCM analyses

All DCM analyses were carried out using DCM8 in SPM8. We focused on the endogenous connectivity (averaged over all correct articulation trials) of the DCM models and assessed the posterior probabilities of the connection parameters using Bayesian inversion by means of expectation and maximisation (EM) (Friston et al., 2003). We do not report modulatory connections in the patient because they were not significant at the single subject level and substantial conclusions about the reading network can be drawn on the basis of the endogenous variability (for a similar rationale see (Abutalebi, Rosa, Tettamanti, Green, & Cappa, 2009). However, the modulatory parameters are reported for the group of 29 healthy controls to test whether connectivity of the different pathways varied for word reading relative to other conditions.

### 2.16 Bayesian model selection (BMS)

To select the most plausible models, we used a Bayesian model selection (BMS) procedure as implemented in SPM8. Model evidence was assessed using the robust and sensitive negative Free energy (F) criterion (Stephan, Penny, Daunizeau, Moran, & Friston, 2009). This criterion points to the optimal compromise between the accuracy and complexity of a given model and provides an approximation for the complexity term that takes into account the interdependency between the estimated parameters and how far their posterior values diverged from their priors.

In the patient, we used a fixed-effect implementation of BMS. In controls, after estimating all models and their evidence (the negative Free energy expressed here as a log-evidence), we computed the group evidence of all models over 29 subjects using the BMS procedure. We used a hierarchical Bayesian approach to ensure that the BMS at the group level was not adversely affected by outliers (Stephan et al., 2009). This random-effects BMS approach quantifies, in the context of a group of subjects, how likely it is that a specific model generated the data of a subject chosen at random (from our 29 subjects). For the group evidence of a given model (Stephan et al., 2009) we used the exceedance probability (*xp*). This probability reflects the evidence that a particular model is more likely than any other model given the group data. For a similar procedure see (Seghier et al., 2011; Seghier & Price, 2010). Because all posterior probabilities should sum to one, the group evidence of a given model depends on the number of models tested. The model with the highest evidence is considered to be the winning model.

#### 2.16.1 Bayesian model averaging (BMA)

Bayesian model averaging (BMA) was then applied over the entire model space to make inferences on connectivity parameters (Penny et al., 2010). BMA can assess the full posterior density on parameters where the contribution of each model to the mean effect is weighted by its evidence (Penny et al., 2010). Therefore, models with the highest evidence make the largest contribution whilst the contribution of models with weak evidence is minimised. BMA is suitable in our context since the model evidence may vary between the patient and controls when sampling the same DCM model space (see discussion in (Seghier et al., 2010). In addition, this model averaging was restricted within each subject to generate within-subject densities for computing posterior means of connectivity parameters for each subject (for a similar procedure, see (Seghier et al., 2011). The significance of each connectivity parameter (endogenous or modulatory) is assessed by the fraction of samples in the posterior density that have the same sign as the posterior mean (posterior densities are sampled with 10,000 data points, i.e., (Penny et al., 2010). Significant effects at each individual connection are reported at a posterior probability threshold of 0.90.

## 3 Results

### 3.1 DCM analyses of the patient data

#### 3.1.1 Step 1: Which visual processing area is the input to the rest of the reading system in the patient?

The comparison of eight different 4 region models (left vOCC, left dOCC, right vOCC, right dOCC) which differed from one another in the location of the input region (Fig. 4) found strong evidence that left vOCC was the input region (the posterior probability for this model was 0.89, a value greater than the sum of the evidence for all other models; see Fig. 4). All further DCM analyses with left hemisphere regions in the patient therefore specified left vOCC as the driving region.

#### 3.1.2 Step 2: Which set of connections best explains the patient’s data in the 5 region left hemisphere model?

**Fig. 5:**
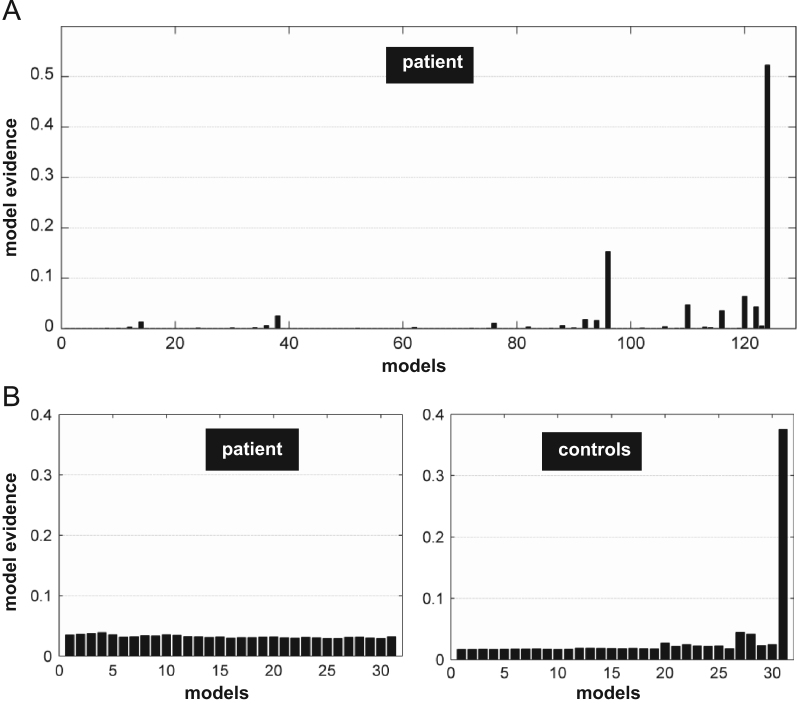
(A) BMS analysis across the 124 possible models with 5 regions in the patient that varied in the number of connections from vOCC to vPM/M through either STS or PT. The best model is the one with the highest posterior model probability. (B) BMS over the 31 models with 6 regions in the patient (left) and controls (right) that connect LvOT to the five left hemisphere regions.

BMS over all possible 124 models in the patient revealed a winning model in which both STS and PT were connected to vOCC, vPM and M (see Fig. 5A). The evidence for this single model (posterior model probability=0.52) was higher than the sum of the evidence for all remaining 123 models.

#### 3.1.3 Step 3: How does evidence change when LvOT is connected to the five left hemisphere region model in the patient?

We found that, as the number of connections with LvOT increased, the model evidence decreased (*r*=−0.89, *p*<0.001) with very low evidence for any of the models that included connections to LvOT (model evidence varied from 0.029 to 0.038, Fig. 5B). As expected, this suggests that connections to LvOT were adding noise to the models rather than accounting for the data. With respect to individual connections (averaged over all 31 models), all endogenous connectivity from the vOCC to M and vPM via STS and PT was strong in the patient with very weak (nonsignificant) connections to and from LvOT (see values in Table 2). These results are consistent with a functionally disabled LvOT.

#### 3.1.4 Step 4: Does the right hemisphere contribute in the patient?

We found no significant evidence (at *p*>0.9) for connectivity among any pairs of right-hemisphere regions (Model A) even when activity from right GPe was included (Model B). The strongest connections involving the right hemisphere regions were coming from their homotopic regions in the left hemisphere (Model C), in particular left to right vOCC (*p*=0.95), left to right STS (*p*=0.99), and left to right PT (*p*=0.99). This suggests that the right hemisphere activation in the patient was potentially driven by the left hemisphere activation.

#### 3.1.5 Summary of DCM in the patient

The 4-step DCM analysis in the patient data established that (1) left vOCC was the driving input region; (2) both STS and PT were involved in linking visual information from left vOCC to the articulation areas in the motor cortex; (3) LvOT did not contribute to the reading network; (4) right hemisphere activation was significantly influenced by activation in the left hemisphere homotopic regions, with insignificant inter-regional connections within the right hemisphere.

### 3.2 DCM analyses of control data

Evidence strongly favoured LvOT connectivity with all other regions when the 31 models with LvOT were compared (*xp*=0.99, see Model 31 in Fig. 5B). This contrasts with the same analysis in the patient (Step 3 above) where very low model evidence was found for all models with LvOT connections (see above). These results are consistent with our a priori assumption that LvOT was contributing in the controls but not in the patient.

**Fig. 6:**
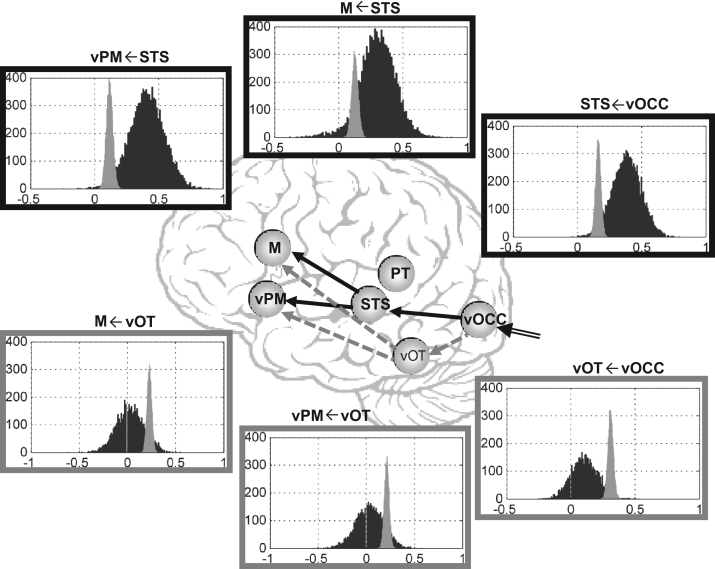
Differences in endogenous connectivity between the patient and controls as assessed using the BMA tool. Connections where the patient increased (or decreased) the connectivity relative to the controls are shown in solid black arrows (or dashed gray arrows). The distribution of the posterior means of each connectivity parameter is illustrated by the histograms for the patient (black distribution) and controls (gray distribution).

A direct comparison of all endogenous connection strengths in controls and the patient revealed significantly stronger connectivity for controls than the patient in all LvOT connections. Significantly stronger connectivity for the patient relative to controls was seen from the vOCC→STS and from the STS→vPM/M (see BMA results in Fig. 6 and Table 2). This dissociation suggests that the patient was relying more on the STS pathway(s) in the context of a dysfunctional LvOT pathway. However, there was also strong evidence that controls were using the vOCC→STS→vPM/M pathway since (a) the vOCC→STS and STS→vPM/M connections were significantly modulated by naming and reading (Table 3); (b) the connection strength from vOCC→STS was highly correlated with the connection strength from STS→vPM/M on both the endogenous and modulatory parameters (*r*=0.72 to *r*=0.93), a finding consistent with a reading pathway from vOCC to vPM/M; and (c) this reading pathway was observed despite the failure of the naming and reading conditions to modulate the STS→LvOT and LvOT→STS connections. When the modulatory effect of reading was compared to the modulatory effect of naming the only significant difference was on the connection strength from vOCC→LvOT (stronger for naming than reading) and from LvOT→vPM and PT→vPM (stronger for reading than naming, Table 3). In summary, although LvOT was significantly involved in controls, strong evidence for a reading-without-LvOT pathway involving STS (Fig. 6) was also found. This is consistent with the findings of Richardson et al. (2010). Below we assessed the independence of the reading-without-LvOT pathway from the reading-with-LvOT pathway.

### 3.3 Inter-subject variability in the reading pathways used by controls

**Fig. 7:**
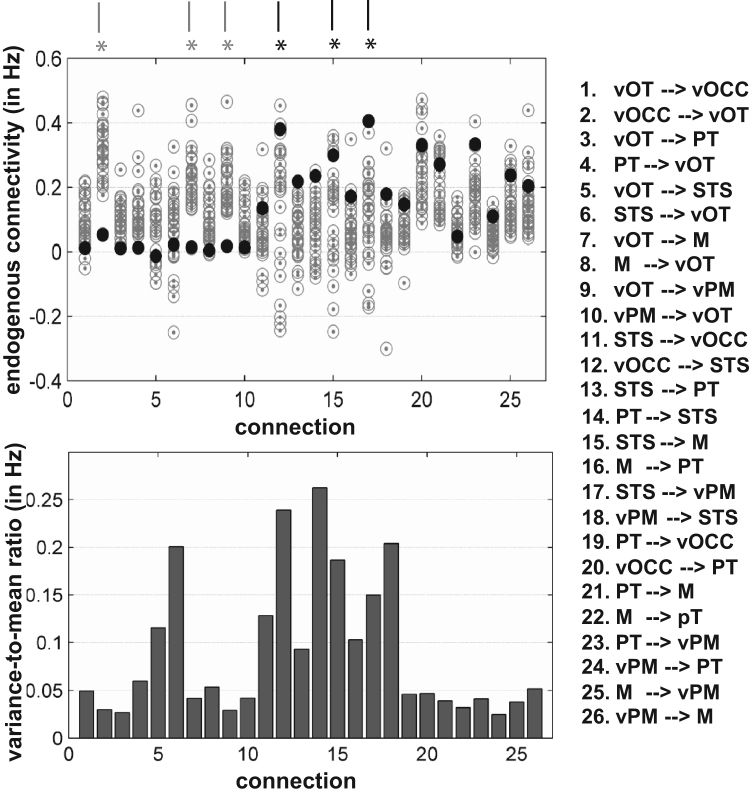
(Top) Illustrates the inter-individual variability in endogenous connectivity across our 29 subjects. Each dot (gray circle) illustrated one healthy subject and the closed circles (black) represented the patient connectivity parameters. (Bottom) The bar graph plots the dispersion of each connectivity parameter as expressed by the variance-to-mean ratio. High values signify wide variability across subjects. A list of all connections is provided at the left side of the figures. The connections indicated by stars correspond to the connections where the differences between the patient and controls were statistically significant (i.e., those shown in Fig. 6 and Table 2).

Evidence for wide variability in the inter-regional interactions in our controls (who all activated the six left hemisphere regions of interest) is illustrated in Fig. 7, using the variance-to-mean ratio as a measure of data dispersion (Cox & Lewis, 1966). The highest dispersion was observed on connections to and from STS (see bar graph in Fig. 7).

**Fig. 8:**
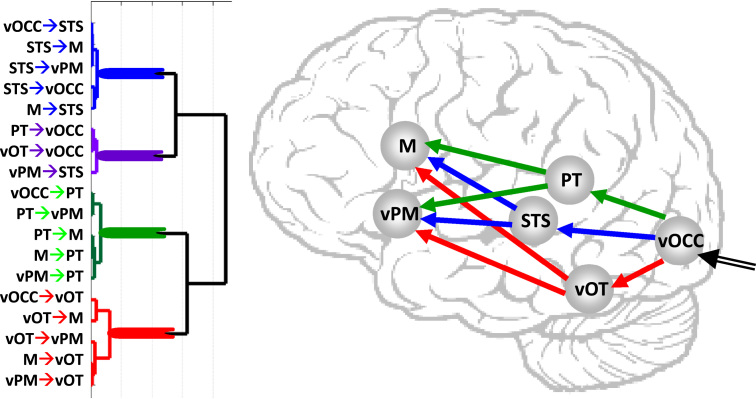
Illustrates the hierarchical clustering of endogenous connectivity over all 29 controls. The dendrogram (left) shows the different branches/clusters that contain highly similar connections. The connections grouped in the same clusters are shown with different colours on a schematic brain.

Hierarchical clustering dissociated the forward connections from vOCC→vPM/M via (i) LvOT, (ii) PT or (iii) STS (see Fig. 8). More specifically, the results showed that when vOCC→STS connectivity was high there was also high STS→vPM (*r*=0.88, *p*<0.001) and STS→M (*r*=0.93, *p*<0.001) connectivity. This is consistent with a functionally connected reading pathway propagating along the vOCC→STS and STS→M/vPM connections. In contrast, this STS pathway was highly dissimilar from the LvOT pathway. This is illustrated in Fig. 8 by the distance between the STS cluster (in blue) and the LvOT cluster (in red) and suggests that the STS pathway would be the most efficient compensatory route when LvOT is damaged since it is least dependent on LvOT. In addition, across control subjects, there were highly significant negative correlations for the modulatory effect of naming and reading between the LvOT→vPM connection and the (i) vOCC→STS connection (*r*=−0.59), (ii) STS→M connection (−0.54), and (iii) STS→vPM connection (−0.47). This suggests that subjects with relatively weaker modulations in the LvOT pathway relied more on the STS pathway. Last but not least, we found no evidence indicating that differential reliance on the STS or LvOT pathway impacted performance for any of our relatively easy in-scanner tasks. For instance, one skilled reader (female, 24 years old) who had very weak connectivity from the vOCC→LvOT (0.05 Hz) but strong connectivity on vOCC→STS (0.38 Hz) connection performed at ceiling level on all our tasks.

## 4 Discussion

This study investigated how accurate identification of rapidly presented words can succeed without LvOT. Our hypothesis was generated from a patient with left occipito-temporal damage and tested in a group of healthy controls who showed variability in the degree to which LvOT was activated during reading. Our functional connectivity analysis of brain activation in the patient while she successfully read aloud short familiar words under speeded conditions demonstrated activation in a reading pathway involving the left STS but not LvOT. This suggests that the left STS pathway can support accurate reading of rapidly presented words. However, as discussed below, we are not claiming that the STS pathway is supporting normal reading because out of scanner testing indicated that the patient’s reading was slower than normal, with a word length effect of 280 ms per letter. Nevertheless, she was able to read most familiar words within 2 s irrespective of their length (Fig. 2).

The STS pathway was also observed in healthy skilled readers and was found to be dissociable from the LvOT pathway. This finding is in line with previous studies that demonstrate how skilled reading can be sustained by multiple neural pathways (e.g., Ben-Shachar, Dougherty, & Wandell, 2007; Ischebeck et al., 2004; Jobard, Vigneau, Simon, & Tzourio-Mazoyer, 2011; Kherif, Josse, Seghier, & Price, 2009; Rosazza, Cai, Minati, Paulignan, & Nazir, 2009; Schlaggar & McCandliss, 2007; Seghier, Lee, Schofield, Ellis, & Price, 2008; Seghier & Price, 2010) that may dissociate earlier than LvOT (Iwata, 1984; Levy et al., 2009; Richardson et al., 2011; Sakurai, 2004) with activation in the STS and other temporal lobe regions correlating with reading skill (Welcome & Joanisse, 2012). Likewise, other recent fMRI studies of patients with vOT damage have identified alternative neural pathways (that do not involve vOT) during covert face recognition (Valdés-Sosa et al., 2011) and number processing (Cappelletti, Leff, & Price, 2012).

The novel contribution of our study is to show how rapid word identification can be supported in the absence of LvOT in a patient with left occipito-temporal damage and how the same reading-without-LvOT pathway is activated by skilled readers, particularly when activation in the LvOT pathway is low. This stresses the importance of mapping alternative processing pathways for the same task in healthy controls (i.e., accounting for degeneracy, see (Price & Friston, 2002)).

Our systems level approach for characterising the impact of damage on a given system/function (Bassett & Bullmore, 2009; He, Shulman, Snyder, & Corbetta, 2007; Rowe, 2010; Seghier et al., 2010; Sharp, Turkheimer, Bose, Scott, & Wise, 2010; Westlake & Nagarajan, 2011) contrasts with all previous neuroimaging case reports of patients with LvOT damage during reading (e.g., Cohen et al., 2004; Cohen et al., 2004; Cohen et al., 2003; Gaillard et al., 2006; Henry et al., 2005; Hillis et al., 2005; Ino et al., 2008; Leff et al., 2001; Michel, 2008; Newhart, Ken, Kleinman, Heidler-Gary, & Hillis, 2007; Philipose et al., 2007; Pyun et al., 2007; Sakurai, 2004; Sakurai et al., 2000; Tsapkini & Rapp, 2010; Tsapkini et al., 2011). Specifically, by characterizing the impact of LvOT damage at the system level we were able to show how activated areas interacted with one another to support residual reading in our patient. This led to novel hypotheses that could be tested in healthy skilled readers. Furthermore, by using recent Bayesian techniques (Penny et al., 2010), we ensured optimal group inferences when comparing the patient and controls at each inter-regional connection within the identified reading system. Using this systematic approach, we were able to demonstrate that, compared to controls, the patient had stronger connectivity in a reading-without-LvOT pathway that involved STS. Thus, the reading-without-LvOT pathway was most activated when LvOT was damaged but was also involved in healthy controls, particularly when connectivity in the LvOT pathway was low.

For the following reasons, we propose that the left STS reading pathway is most likely to be involved in the integration of semantics with phonology. First, the location of the left STS region at [*x*=−64 *y*=−28 *z*=−2] is between two temporal subsystems (Binder, Desai, Graves, & Conant, 2009; Hickok & Poeppel, 2007), a posterior-dorsal temporal subsystem that is likely to be phonological in nature and an anterior–ventral temporal subsystem that is likely to be semantic in nature. Second, our fMRI results showed that the left STS was significantly activated during reading aloud, object naming and semantic decisions on written words. Third, our DCM findings showed that all connections to and from left STS were similarly modulated by word reading and object naming (Table 3) which both involve the translation of semantics to phonology, albeit differently. Fourth, a recent MEG study of written word processing (Kujala, Vartiainen, Laaksonen, & Salmelin, 2012) showed a significant overlap between phonological and semantic priming effects in the same left STS area [at *x*=−61 *y*=−26 *z*=3]. Fifth, a recent fMRI study of normal readers, reading silently (Jobard et al., 2011), found increased left STS activation in less proficient readers (see Fig. 3B of (Jobard et al., 2011)). This is consistent with high left STS activation in our patient with left vOT damage and impaired reading. Sixth, in the same way, fMRI activation in left STS significantly correlated with word length or naming latencies (Wilson, Isenberg, & Hickok, 2009), and positively correlated with reaction times during reading aloud monosyllabic English words (Graves, Desai, Humphries, Seidenberg, & Binder, 2010). In summary, we speculate that successful reading without LvOT increases the demands on the translation of semantics to phonology in the left STS.

Clearly, the STS reading pathway was not sufficient to sustain normal reading in the patient because the patient’s reading was slower and more error prone than the controls. On the other hand, some of the control subjects could read rapidly and accurately despite low connectivity in the LvOT pathway and high connectivity in the STS pathway. This suggests that these controls had processing power that was not available to the patient. One possibility is that even low LvOT activation boosts reading ability in the controls. Alternatively, there may be other pathways in the vicinity of LvOT that were damaged in the patient but used by controls. Indeed, it is important to keep in mind that (i) our patient’s lesion was not restricted to LvOT but included the surrounding white matter pathways that have been shown to be critical in language and object recognition in general (Catani, Jones, Donato, & Ffytche, 2003; Epelbaum et al., 2008; Saur et al., 2008; Yeatman, Rauschecker, & Wandell, in press), (ii) the loss of these white matter pathways will disrupt the bottom-up and top-down interactions between LvOT and STS during word recognition (Kujala et al., 2012) and (iii) it is not unusual that recovery continues even decades after stroke (e.g., Berthier et al., 2011; Naeser et al., 1998; Price, Seghier, & Leff, 2010; Smania et al., 2010). The functional capacity of the alternative pathways, tested here at 4 years after stroke, may therefore improve with time. Future studies are required to address these issues.

Two unexpected findings also need to be addressed. First, our results in the patient did not indicate abnormal involvement of the superior parietal lobule, even though previous studies have shown that this region has strong functional connectivity with the LvOT that increases with reading skill (Vogel, Miezin, Petersen, & Schlaggar, 2012), and is involved in compensatory mechanisms after LvOT damage (see cases in Henry et al., 2005; Ino et al., 2008). Valdois et al., (2006) have suggested that the superior parietal lobule may sustain visuo-attentional analysis required during analytical reading (Valdois et al., 2006), a strategy that might be used by our patient when reading slowly. Indeed, our patient showed significant activation in bilateral superior parietal lobule for reading aloud relative to fixation (Fig. 3A); however superior parietal activation was not significantly greater in the patient than controls (see Table 1) and was thus excluded from our DCM analysis. We did not find significantly more superior parietal activation in our patient (or controls) for reading than saying “123” to unfamiliar Greek letters (*p*<0.001 uncorrected), even though saying “123” was unrelated to the visual stimuli and thus required less visual attention than reading. This is consistent with previous reports showing that superior parietal activation is more likely to increase when the task is unusually challenging, for example, when reading polysyllabic pseudo-words (Valdois et al., 2006), degraded words (Cohen, Dehaene, Vinckier, Jobert, & Montavont, 2008), mixed-case words (Mayall, Humphreys, Mechelli, Olson, & Price, 2001), vertically presented words (Rosazza et al., 2009), and longer words and nonwords (Church, Balota, Petersen, & Schlaggar, 2011; Schurz et al., 2010). In this context, we hypothesize that the superior parietal cortices would be more involved when the patient was reading longer words or pseudowords. We are therefore proposing that the STS pathway is more involved in reading short words, whereas the superior parietal cortices will be more involved in reading longer words.

A second issue concerns the lack of right hemisphere involvement in the connectivity pattern of our patient. Previous studies have suggested that the contribution of the right hemisphere is important to letter by letter reading after LvOT damage (e.g., Cohen et al., 2004; Cohen et al., 2004; Gaillard et al., 2006; Leff et al., 2001; Pyun et al., 2007). In our patient, who was discouraged from using an overt letter by letter reading strategy, right hemisphere activation, including that in right vOT was within the normal range (Table 1), except in the right occipital cortex and globus pallidus (Fig. S4 of the Supplementary material). There are several different reasons that our results diverge from previous findings. First, our patient was investigated using an fMRI paradigm that focused on the relatively rapid presentation of short familiar words. This contrasts to the paradigms that have used longer, less frequent words under less constrained conditions (Cohen et al., 2004). Second, our fMRI data were acquired 4 years after stroke and we thus cannot rule out a possible compensatory role of the right hemisphere in the first year after stroke, a compensatory role that may have shifted later to the left hemisphere to sustain more stable long-term recovery. Third, the contribution of the right hemisphere regions in our connectivity model was not tested under optimal conditions. This is because the posterior means of the connectivity parameters are conditional to the model (Daunizeau, David, & Stephan, 2011), and here we could not search the structure of the optimal model in a systematic way because of the large number of regions involved in Step 4 of the DCM analysis in our patient; see (Seghier et al., 2010) for a review on previous DCM studies that used one single model in patients. Although we cannot rule out the importance of right hemisphere regions, our DCM findings show that the most significant inter-regional interactions involved left rather than right hemisphere regions.

We have so far discussed reading pathways that involve LvOT, STS, the superior parietal cortices and the right hemisphere. The importance of the inferior parietal cortices in sublexical spelling to sound conversion has also been highlighted in a study of reading in patients who have surface dyslexia following damage (atrophy) to anterior and inferior temporal regions (Hodges & Patterson, 2007; Wilson & Brambati et al., 2009). These patients have deteriorated semantic knowledge, profound anomia and show an over reliance on sublexical spelling to sound conversion when reading words with irregular spellings (Woollams, Ralph, Plaut, & Patterson, 2007). The increase in inferior parietal activation therefore suggests that these areas are involved in a sublexical reading strategy (Wilson & Brambati et al., 2009). Interestingly, the inferior parietal activation was observed in the absence of activation in either LvOT or STS (Wilson & Brambati et al., 2009). Together with other results, including our own, this suggests that low activation in LvOT can be overcome in different ways using: STS (in our patient with LvOT damage); inferior parietal activation (following anterior temporal lobe damage); superior parietal activation (Ino et al., 2008); or right vOT (Cohen et al., 2004).

Our work has theoretical implications for understanding the neural systems that support reading. Current reading models typically assume that LvOT plays an essential role in reading by linking visual inputs to the language system (see recent reviews in (Dehaene & Cohen, 2011; Price & Devlin, 2011; Wandell, 2011). However, our data suggest that, although loss of LvOT impairs reading, it is still possible to have relatively rapid access to the left hemisphere language system for short familiar words. This is in line with recent evidence (Richardson et al., 2011) that activation during silent reading in healthy skilled readers is better explained by models that connect the occipital cortex to the language system via two pathways (one with and one without LvOT) rather than one pathway (either with or without LvOT). However, the current study goes beyond this previous observation by demonstrating that (1) the reading-without-LvOT pathway is sufficient to support some degree of rapid word identification when the reading-with-LvOT pathway is damaged; (2) in healthy skilled readers, modulation of functional connectivity during naming and reading in the reading-without-LvOT pathway is stronger when there is weaker modulation of functional connectivity in the reading-with-LvOT pathway; and (3) the reading without LvOT pathway involves an STS area that is associated with semantic to phonological integration. Future studies are now needed to determine whether the STS area activated in our patient and controls during reading aloud and the more posterior STS area activated during silent reading in Richardson et al. (2011) are parts of the same or different pathways.

Our findings also have implications for cognitive models of reading. Previous neuropsychological and computational reading models have dissociated different semantic and non-semantic reading routes (Coltheart, Curtis, Atkins, & Haller, 1993; Plaut, McClelland, Seidenberg, & Patterson, 1996; Seidenberg & McClelland, 1989). For instance, many other functional imaging studies have associated the semantic reading route with a ventral visual processing stream that includes LvOT (Price & Mechelli, 2005; Pugh et al., 2001) and contrasted this to nonsemantic routes that involve more dorsal temporal and temporo-parietal regions (Pugh et al., 2001; Sun, Yang, Desroches, Liu, & Peng, 2011; Vigneau, Jobard, Mazoyer, & Tzourio-Mazoyer, 2005). By dissociating the LvOT reading pathway from the STS reading pathway, our results further support the existence of more than one reading route. Moreover, we have suggested above that (i) the superior parietal cortices are involved in an additional pathway that is involved in attention demanding contexts; and (ii) the inferior parietal cortices are involved in spelling to sound translation. The independence of each of these reading pathways on behaviour warrants further investigation.

It is worth noting that the different reading pathways (including those involving LvOT or STS) are not necessarily mutually exclusive but are likely to operate in parallel and interact in different time windows during reading (e.g., Mainy et al., 2008; Pammer et al., 2004; Wilson et al., 2007). The different pathways may also involve overlapping parts of the same white matter tracts that anatomically link posterior (occipital) to anterior (frontal) language regions (Glasser & Rilling, 2008). Likewise, the different pathways are likely to overlap in many different cortical regions (Hagmann et al., 2008; He et al., 2009; Turken & Dronkers, 2011). For instance, resting-state functional connectivity with LvOT and STS as seed regions identified different networks that strongly overlapped at posterior temporal (*x*=−48 *y*=−62 *z*=4) and frontal (*x*=−54 *y*=6 *z*=18) regions in skilled readers (Koyama et al., 2010).

With respect to the clinical implications of our work, we have emphasized that the STS pathway is not the only possible alternative pathway to the LvOT pathway. The involvement of one or more of the alternative pathways, following LvOT damage, may depend on several factors, including: the extent and exact location of the lesion, the amount of preserved white matter tracts, the age and health of the patient, the influence of behavioral interventions and normal inter-subject variability in the preferred reading strategy. This hypothesis explains why several other studies have shown different activation patterns in the left and right hemisphere in patients with LvOT damage who continued to read, albeit at a slow rate (e.g., Cohen et al., 2004; Cohen et al., 2004; Cohen et al., 2003; Gaillard et al., 2006; Henry et al., 2005; Ino et al., 2008; Leff et al., 2001; Newhart et al., 2007; Pyun et al., 2007; Sakurai, 2004; Sakurai et al., 2000; Tsapkini et al., 2011).

At present, there are few fMRI studies of patients with LvOT damage; and the methodological approach has differed over studies. In this sense, our study adds to the diversity of available data. However, there are also some consistent findings. For example, the existence of multiple reading pathways has previously been emphasized on both theoretical and clinical grounds, even though the exact anatomy and function of these pathways remains unclear. Although the literature is currently flooded with inconsistency in both neuropsychological case reports and fMRI results, the development of new analysis tools and access to large databases are now providing a means to unravel the anatomical and psychological variables that influence reading.

In summary, our results demonstrate the existence of a reading pathway involving left STS in the absence of LvOT. This pathway was sufficient to support identification of familiar words in a patient with extensive LvOT damage. It was also found to be independent of the LvOT reading pathway in healthy skilled readers. Future studies are motivated to investigate how this STS pathway interacts with other left or right fronto-parietal areas that were not included in our DCM models. It will also be interesting to investigate how the STS pathway interacts under other tasks/contexts and in other populations with different reading difficulties. The characterisation of all potential reading pathways would add more flexibility to current reading models (Plaut, 1996). Understanding the effect of damage to these different pathways may also help to tailor more efficient therapy (Hillis, 1993).

## Figures and Tables

**Table 1:** List of activations (MNI coordinates and *Z* scores) during reading aloud relative to fixation in the patient and the patient compared to controls (at *p*<0.05 FWE-corrected). The *Z* scores for the patient>controls are reported from the second-level analysis for reading relative to fixation. For controls, up to three local maxima for each cluster are listed. n.s.=not significant at *p*<0.05 uncorrected; *Pt*=patient *Ct*=controls. Other regions activated in the patient in other contrasts are listed in Table S2 of the Supplementary material.

Regions	Left hemisphere			Right hemisphere		
	Patient coord.; *Z* score	Controls coord.	*Pt*>*Ct Z* score	Patient coord.; *Z* score	Controls coord.	*Pt*>*Ct Z* score
Ventral occipital cortex (vOCC)	−30 −86 2; 5.1	−26 −92 8	3.8	26 −78 2; 4.9	22 −94 2	4.5
		−22 −94 4			16 92 6	
		−10 −96 8			12 −88 8	
Dorsal occipital cortex (dOCC)	−26 −84 30; 5.6	−22 −94 18	5.3	24 −84 32; 4.3	26 92 16	4.2
		−28 −76 24			30 −72 32	
		−28 −84 18			30 −88 22	
Ventral occipito-temporal cortex (vOT)	n.s.	−40 −76 −10	n.s.	42 −54 −14; 1.9	44 −76 −12	1.9
		−40 −56 −16			44 −62 −16	
		−36 −44 −24			36 −44 −18	
Superior temporal sulcus (STS)	−64 −28 −2; 6.3	−56 −28 2	4.1	68 −32 −4; 5.8	54 −30 4	4.3
		−66 −28 4			64 −26 8	
Planum temporale (PT)	−44 −40 10; 5.1	−52 −40 4	5.6	46 −36 4; 4.1	48 −−36 10	2.7
		−50 −46 14			56 −36 12	
Ventral premotor cortex (vPM)	−56 4 4; 6.6	−62 −4 2	5.7	64 8 0; 5.5	64 0 2	n.s.
		−60 6 0			60 12 0	
Motor cortex (M)	−42 −16 28; 4.6	−46 −14 38	4.7	42 −12 34; 5.9	50 −10 42	2.3
		−54 −6 28			42 −12 36	
		−64 −18 10			54 −16 4	
Globus pallidus (GPe)	n.s.	−24 −8 4	n.s.	22 −12 −2; 4.0	n.s.	4.8
		−26 −14 −2				

**Table 2:** The average strength of endogenous connectivity parameters (in Hz) for the patient and over our 29 controls using BMA tool over the 31 DCM models. Values in bold are significant at a posterior probability >0.90 and values in *italics* represent connectivity parameters that are weaker in the patient than controls. *Pt*=patient; *Ct*=controls.

Patient						
	vOCC	PT	STS	vOT	M	vPM
vOCC	–	0.15	0.14	0.01	–	–
PT	**0.33**	–	**0.22**	0.01	0.05	0.11
STS	**0.38**	**0.24**	–	−0.02	0.17	**0.18**
vOT	0.05	0.01	0.02	–	0.00	0.01
M	–	**0.27**	**0.30**	0.01	–	**0.20**
vPM	–	**0.33**	**0.41**	0.02	**0.24**	–

Controls						
	vOCC	PT	STS	vOT	M	vPM

vOCC	–	**0.08**	**0.06**	**0.10**	–	–
PT	**0.24**	–	**0.06**	**0.10**	**0.07**	**0.06**
STS	**0.16**	**0.10**	–	**0.08**	**0.07**	**0.05**
vOT	**0.31**	**0.12**	**0.07**	–	**0.10**	**0.09**
M	–	**0.20**	**0.13**	**0.23**	–	**0.15**
vPM	–	**0.17**	**0.12**	**0.21**	**0.15**	–

Patient vs. controls						
	vOCC	PT	STS	vOT	M	vPM

vOCC	–	*Pt*>*Ct*	*Pt*>*Ct*	*Pt*<*Ct*	–	–
	–	*p*=0.62	*p*=0.71	*p=0.84*	–	–
*Pt*	*Pt*>*Ct*	–	*Pt*>*Ct*	*Pt*<*Ct*	*Pt*=*Ct*	*Pt*>*Ct*
	*p*=0.78	–	*p*=0.87	*p=0.85*	*p*=0.50	*p*=0.64
STS	*Pt*>*Ct*	*Pt*>*Ct*	–	*Pt*<*Ct*	*Pt*>*Ct*	*Pt*>*Ct*
	***p*=0.97**	*p*=0.84	–	*p=0.82*	*p*=0.75	*p*=0.83
vOT	*Pt*<*Ct*	*Pt*<*Ct*	*Pt*<*Ct*	–	*Pt*<*Ct*	*Pt*<*Ct*
	***p=0.99***	*p=0.88*	*p=0.79*	–	*p=0.88*	*p=0.85*
M	–	*Pt*>*Ct*	*Pt*>*Ct*	*Pt*<*Ct*	–	*Pt*>*Ct*
	–	*p*=0.68	***p*=0.90**	***p=0.95***	–	*p*=0.66
vPM	–	*Pt*>*Ct*	*Pt*>*Ct*	*Pt*<*Ct*	*Pt*>*Ct*	–
	–	*p*=0.86	***p*=0.98**	***p=0.94***	*p*=0.73	–

**Table 3:** The average strength of modulatory parameters (in Hz) across all our 29 controls using the BMA tool over the 31 DCM models. Values in bold are significant at a posterior probability >0.90.

Naming+reading						
	vOCC	*Pt*	STS	vOT	M	vPM
vOCC	–	−0.01	**−0.03**	−0.01	–	–
PT	**0.07**	–	−0.01	0.00	−0.02	0.01
STS	**0.06**	0.03	–	0.01	0.01	0.00
vOT	**0.12**	**0.04**	0.02	–	0.02	**0.04**
M	–	**0.03**	**0.03**	**0.06**	–	**0.03**
vPM	–	**0.07**	**0.06**	**0.07**	**0.05**	–

Naming−reading						
	vOCC	PT	STS	vOT	M	vPM

vOCC	–	0.02	0.02	0.02	–	–
PT	−0.02	–	−0.01	−0.02	−0.02	−0.02
STS	−0.01	−0.01	–	−0.01	−0.02	−0.02
vOT	**0.06**	0.03	0.01	–	0.03	0.02
M	–	−0.02	−0.01	−0.02	–	−0.01
vPM	–	**−0.04**	−0.02	**−0.03**	**−0.03**	–

## Footnotes

Appendix A Supplementary data associated with this article can be found in the online version at 10.1016/j.neuropsychologia.2012.09.030.
