# Supplementary material for: Reading without the left ventral occipito-temporal cortex
Source: Neuropsychologia. 2012 Dec;50-360(14):3621–35. doi: 10.1016/j.neuropsychologia.2012.09.030 (PMC3524457; doi:10.1016/j.neuropsychologia.2012.09.030)

Table S1: MNI coordinates of the six regions of interest in all our 29 controls.

	vOCC			PT			STS			vOT			M			vPM		
**Sub**	x	y	z	x	y	z	x	y	z	x	y	z	x	y	z	x	y	z
**S1**	-32	-90	-2	-44	-46	10	-60	-28	0	-42	-54	-16	-44	-12	32	-56	8	0
**S2**	-34	-82	2	-44	-40	12	-62	-26	0	-40	-58	-18	-44	-12	32	-60	4	0
**S3**	-30	-92	2	-40	-36	12	-64	-24	-4	-44	-60	-16	-42	-16	34	-54	0	4
**S4**	-30	-88	-2	-46	-36	6	-64	-26	-2	-42	-58	-20	-46	-12	30	-56	2	4
**S5**	-34	-88	6	-46	-36	14	-66	-26	0	-44	-60	-16	-46	-12	30	-58	8	0
**S6**	-34	-88	-4	-44	-36	14	-60	-32	0	-44	-58	-18	-44	-14	32	-60	2	6
**S7**	-34	-90	-6	-42	-36	14	-68	-34	-2	-40	-52	-12	-42	-16	34	-60	0	4
**S8**	-34	-88	-12	-48	-44	8	-64	-24	-4	-40	-56	-20	-46	-12	30	-60	0	2
**S9**	-34	-92	-4	-44	-40	4	-66	-32	-2	-46	-50	-20	-46	-14	32	-60	2	8
**S10**	-34	-90	-4	-44	-40	16	-64	-26	-8	-44	-54	-22	-44	-14	32	-56	4	-2
**S11**	-26	-88	-12	-48	-36	10	-60	-32	-4	-40	-58	-18	-46	-14	32	-58	0	0
**S12**	-32	-86	-8	-48	-38	14	-66	-30	0	-44	-58	-16	-44	-14	32	-60	0	2
**S13**	-28	-90	-4	-42	-44	6	-68	-28	0	-40	-54	-18	-44	-12	32	-60	2	0
**S14**	-36	-90	-8	-40	-40	14	-68	-34	-2	-46	-52	-14	-42	-16	34	-60	2	0
**S15**	-28	-92	-4	-40	-38	14	-64	-30	2	-40	-58	-18	-46	-12	30	-58	6	0
**S16**	-30	-90	-8	-44	-40	16	-60	-26	-6	-42	-56	-20	-42	-16	34	-62	4	4
**S17**	-34	-86	-8	-44	-34	10	-62	-34	-2	-48	-52	-20	-42	-14	32	-56	8	0
**S18**	-34	-88	-4	-42	-36	14	-66	-34	0	-42	-58	-20	-44	-12	32	-62	4	4
**S19**	-34	-92	-12	-42	-36	14	-68	-30	-2	-40	-56	-18	-46	-12	28	-60	2	0
**S20**	-30	-90	-2	-48	-44	12	-66	-34	0	-38	-54	-16	-44	-12	32	-60	4	0
**S21**	-28	-94	-12	-44	-34	10	-60	-30	0	-46	-56	-20	-42	-16	34	-58	8	0
**S22**	-26	-94	-10	-48	-44	12	-60	-28	-2	-40	-58	-18	-42	-16	34	-60	8	2
**S23**	-30	-94	-4	-42	-36	14	-68	-28	0	-38	-54	-16	-44	-14	32	-56	4	-2
**S24**	-28	-92	-12	-44	-46	10	-68	-26	-2	-40	-54	-20	-46	-14	32	-60	8	4
**S25**	-30	-90	-10	-46	-42	8	-64	-36	-4	-50	-54	-16	-42	-16	34	-60	6	2
**S26**	-34	-88	-12	-48	-44	8	-64	-30	2	-40	-54	-20	-46	-14	32	-60	4	0
**S27**	-28	-94	-4	-46	-36	14	-62	-28	-8	-40	-52	-20	-46	-14	32	-62	4	4
**S28**	-24	-90	-8	-48	-42	12	-64	-24	-4	-44	-58	-12	-44	-12	32	-60	6	0
**S29**	-34	-92	-12	-48	-42	6	-62	-32	-6	-44	-54	-22	-44	-12	32	-56	6	4
**Mean**	**-31**	**-90**	**-6**	**-45**	**-40**	**11**	**-64**	**-29**	**-2**	**-42**	**-56**	**-18**	**-44**	**-14**	**32**	**-59**	**4**	**2**
**SD**	3.2	2.8	4.8	2.7	3.7	3.3	2.9	3.5	2.6	3.0	2.7	2.6	1.6	1.6	1.5	2.1	2.8	2.4

Table S2: MNI coordinates of the activated foci in the patient (first-level analysis at p<0.001 uncorrected). The activations during reading aloud are illustrated in Figure 3A and Table 1 of the main manuscript. The activated patterns of the different tasks in the healthy subjects can be found in our previous work (see Josse et al. 2008 and 2010; Seghier et al. 2010 and 2011).

Region	coordinates			Z-score
Semantic matching on pictures & words > perceptual matching				
Left inferior frontal gyrus	-56	20	28	5.28
	-50	20	22	4.49
	-48	6	18	4.89
	-44	28	20	4.42
	-44	28	20	4.42
Right inferior frontal gyrus	44	32	22	4.46
Left angular gurus	-30	-68	30	4.30
	-30	-76	42	4.18
Left precentral gyrus	-42	0	36	3.75
Right middle frontal gyrus	36	-2	38	3.66
	40	4	42	3.32
Left superior parietal lobule	-34	-60	46	3.65
Say "123" > perceptual matching				
Left motor and somatosensory cortex	-42	-14	32	7.80
	-62	-2	10	6.65
	-56	16	-14	6.64
Right motor and somatosensory cortex	44	-10	34	7.18
	60	-8	20	5.57
	68	-6	8	5.40
Left auditory cortex	-38	-30	14	4.84
	-50	-26	8	3.84
Right auditory cortex	48	-16	2	3.44
Right posterior superior temporal sulcus	46	-38	6	4.72
	54	-26	2	3.66
Right middle temporal gyrus	68	-40	2	4.64
	68	-30	0	4.29
Left middle temporal gyrus	-66	-28	-2	4.50
	-66	-20	2	4.47
Left planum temporale	-46	-40	16	4.34
	-52	-40	10	3.97
Left superior temporal gyrus	-48	-18	6	3.75
Left pars opercularis	-48	6	14	3.75
Anterior cingulate cortex	-6	24	20	3.52
Naming and Reading > saying 123				
Right medial superior frontal gyrus	12	58	28	4.66
Left medial superior frontal gyrus	-6	60	28	4.63
Left inferior frontal gurus	-46	28	14	4.14
	-54	22	20	3.27
Right middle frontal gyrus	32	42	32	4.08
Left middle frontal gyrus	-22	46	36	4.03
Anterior cingulate cortex	-2	24	36	3.98

Figure S1: summary of the central field test showing a small bilateral right superior visual field loss which has improved since her first test after stroke (the number of missed stimulations decreased with time after stroke).

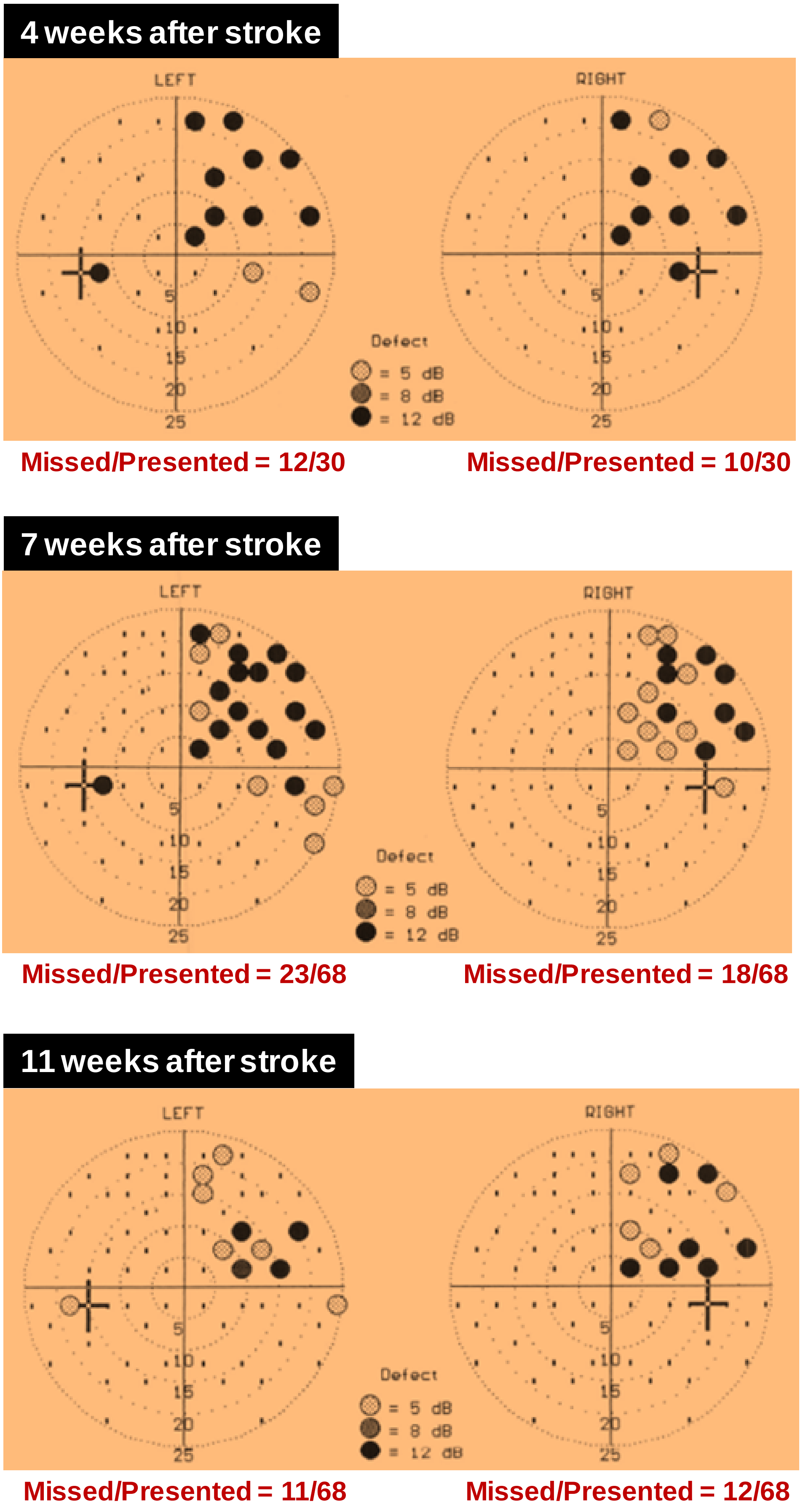

Figure S2: illustrates that the patient's writing was relatively preserved as tested with a picture description task at 5 months after stroke. See for example her spelling of the word "grandfather" and some self-corrections for the words "piece" and "apples". A very few orthographic errors were made; e.g. "triming" and "delifor".

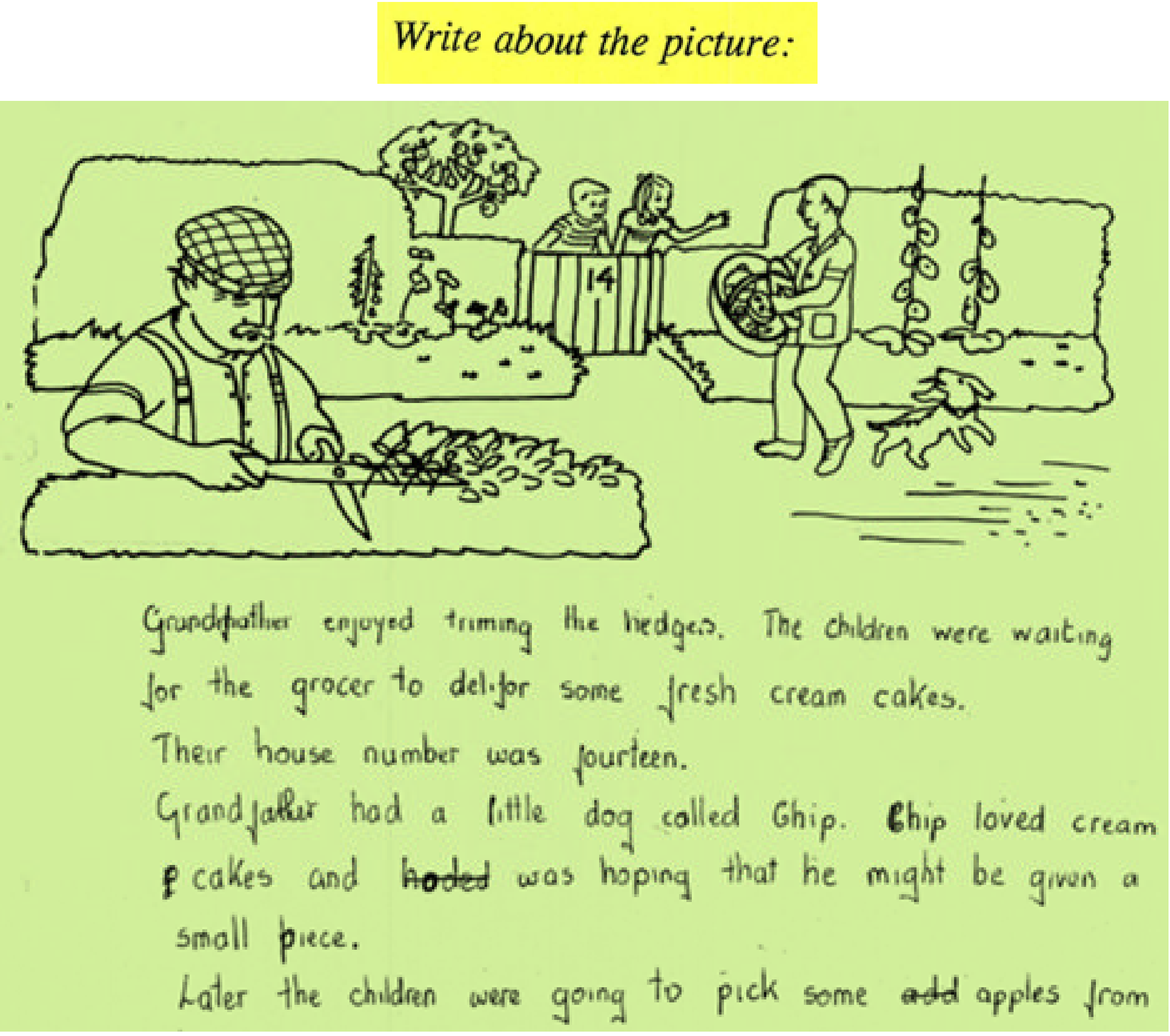

Figure S3: illustrates that her comprehension of written familiar words was relatively preserved as tested with a semantic association task.

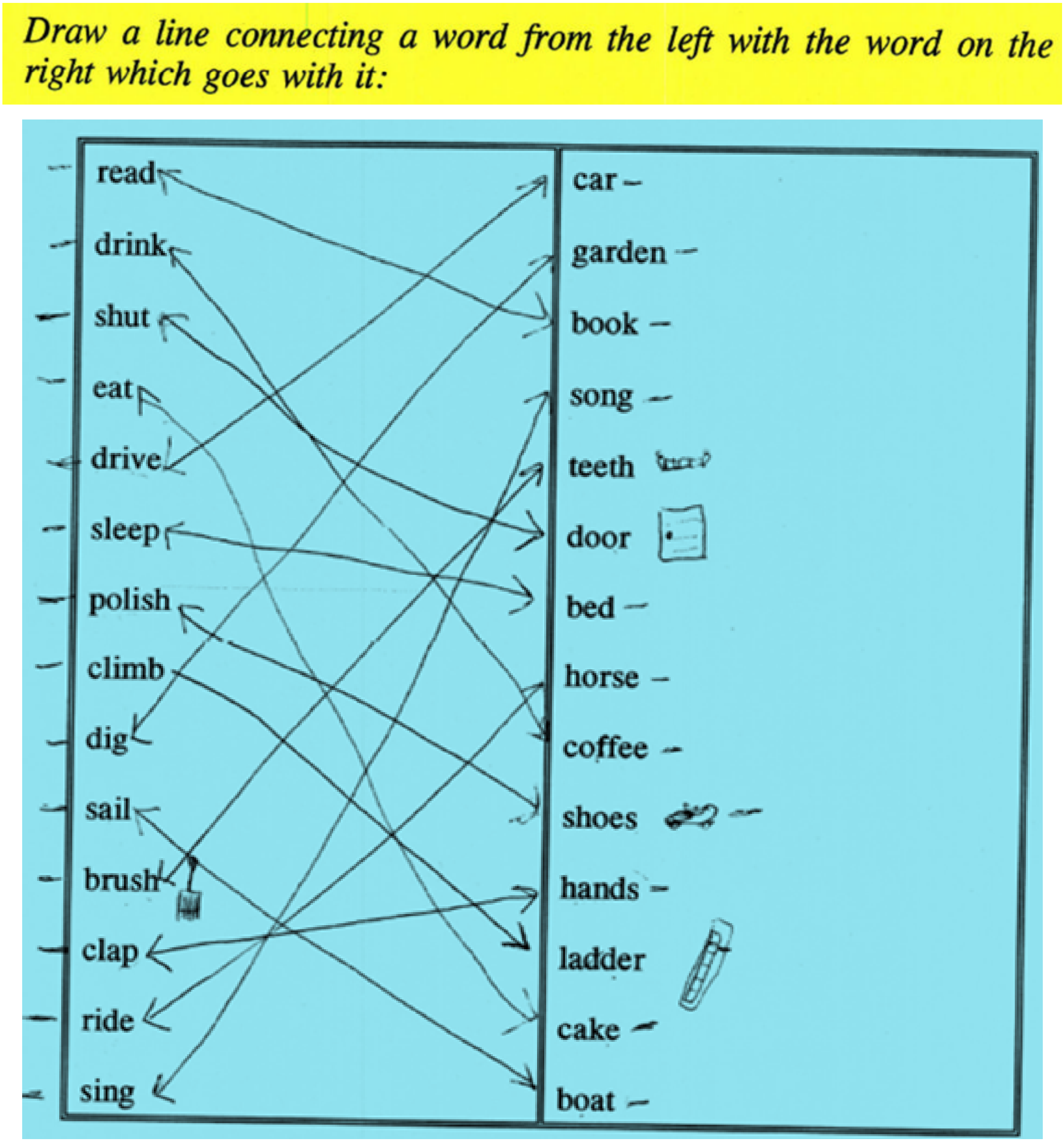

Figure S4: illustrates the activations in controls (left column) and the patient (right column) during visual processing (regions in blue that were common to all 8 conditions), articulation (regions in red that were more activated during naming, reading and saying "123" to unfamiliar stimuli than semantic and perceptual conditions), and semantic (regions in yellow that were more activated for familiar than unfamiliar stimuli). Regions that were significantly higher in the patient during reading aloud compared to controls (at p<0.05 FWE-corrected) are indicated by a white outline in the right column.

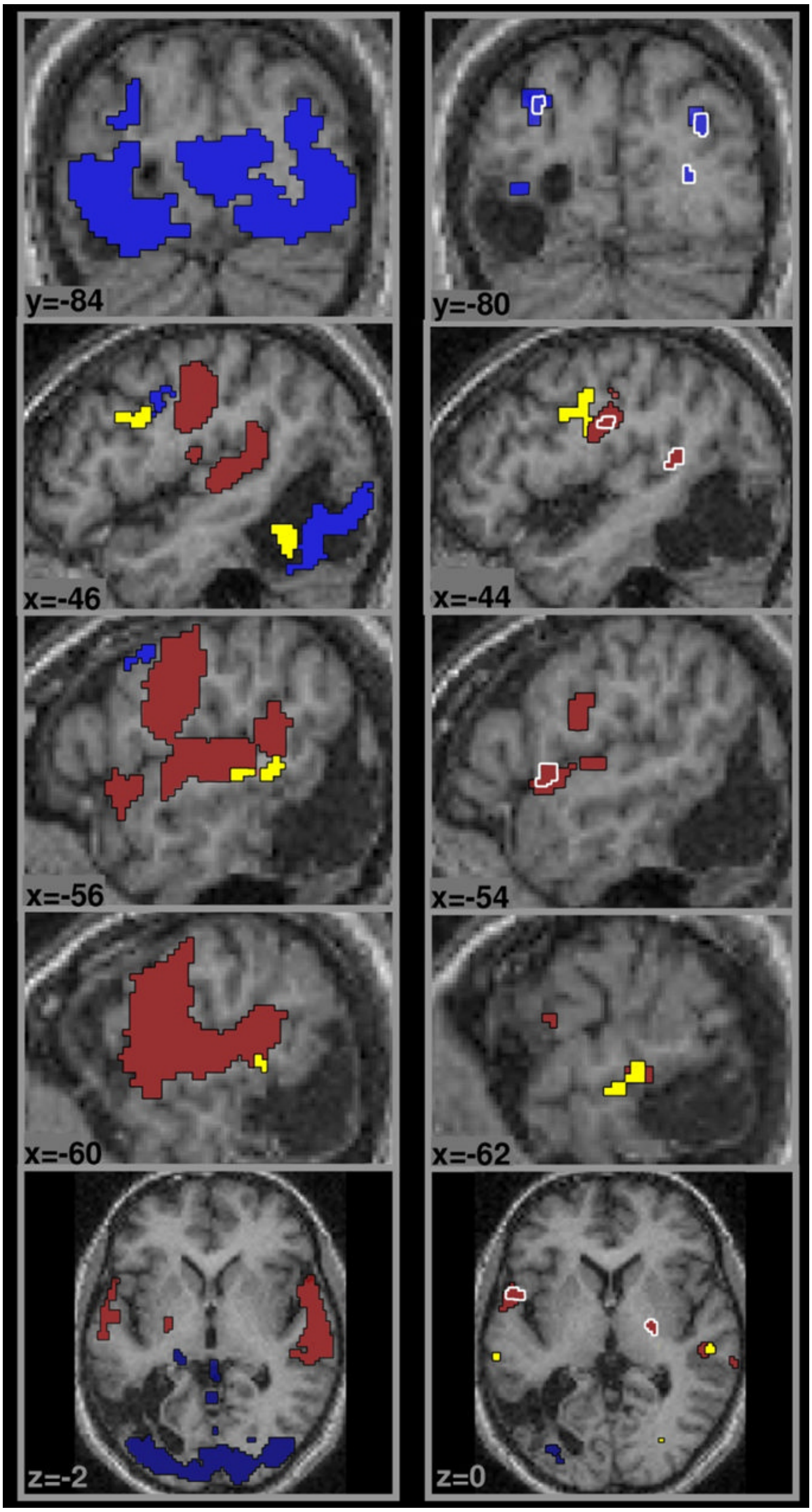

Figure S5: plots of the effect size in each of the 5 ROIs in the patient (regions included in the DCM analyses). For a full list of coordinates see Table 1 of the main manuscript. The x-axis represents the different tasks: 1 = naming pictures aloud, 2 = reading aloud, 3 = saying 1,2,3 to meaningless Greek letter strings and nonobjects, 4 = semantic decisions on pictures of objects, 5 = semantic decisions on written words, 6 = perceptual matching on unfamiliar Greek symbols and nonobjects. Only correct responses were considered here during computing the average effect size, which may explain the weak activations in the articulatory regions vPM and M during object naming (her accuracy during naming was less than 50%). vOCC = ventral occipital cortex, STS = superior temporal cortex, PT = planum temporale, vPM = ventral premotor cortex, M = motor cortex.

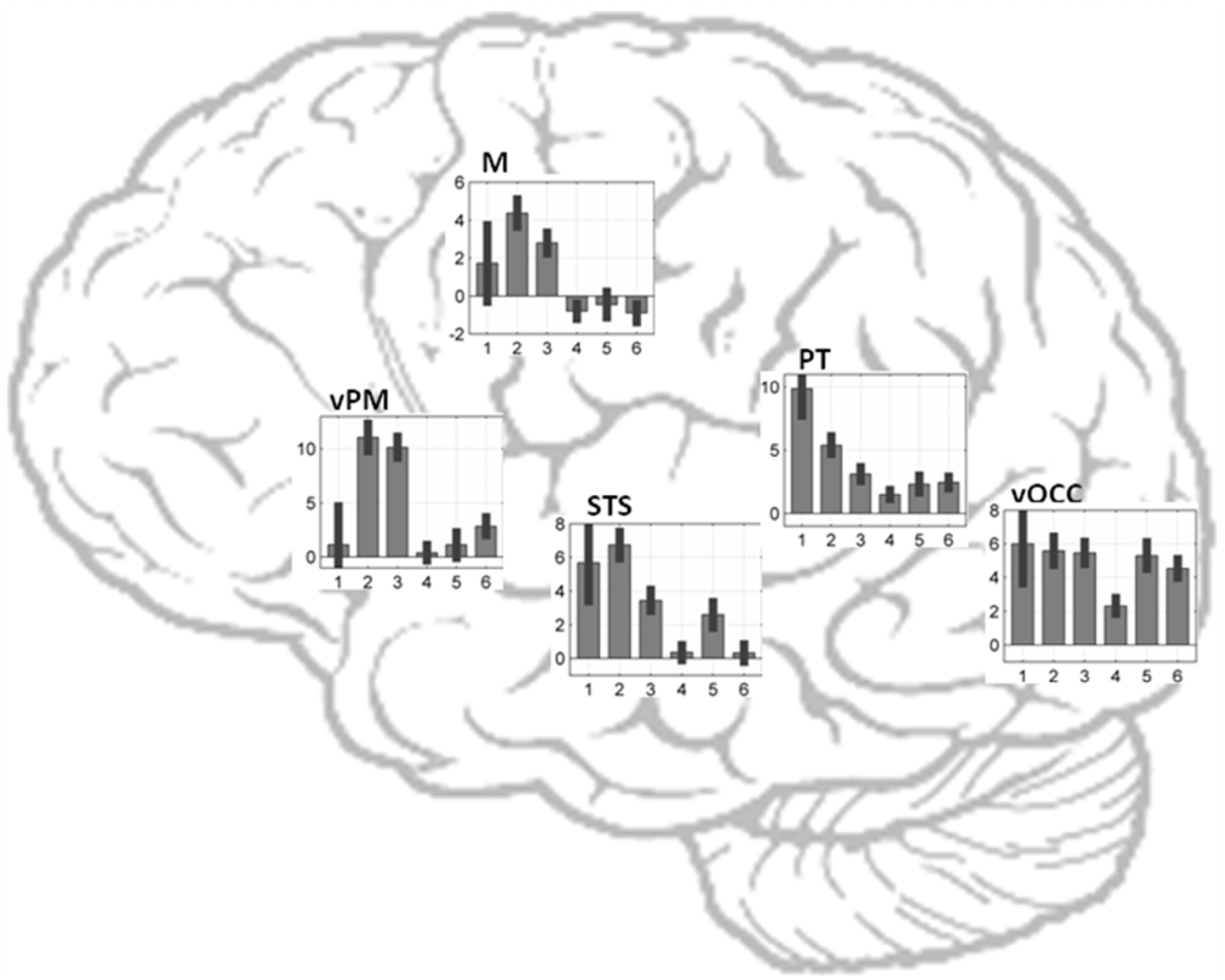

Supplement: Supplementary file 1 — Supplementary data [file mmc1.pdf]
